# Lymphatic topology reveals a novel intranodal lympho‐venous shunt

**DOI:** 10.1002/path.70032

**Published:** 2026-02-04

**Authors:** Ariunbuyan Sukhbaatar, Radhika Mishra, Akira Nakamura, Shiro Mori, Tsuyoshi Sugiura, Tetsuya Kodama

**Affiliations:** ^1^ Division of Oral and Maxillofacial Oncology and Surgical Sciences, Graduate School of Dentistry Tohoku University Sendai Japan; ^2^ Laboratory of Biomedical Engineering for Cancer, Graduate School of Biomedical Engineering Tohoku University Sendai Japan; ^3^ Biomedical Engineering Cancer Research Center, Graduate School of Biomedical Engineering Tohoku University Sendai Japan; ^4^ Professor Emeritus, Department of Immunology Tohoku Medical and Pharmaceutical University Sendai Japan

**Keywords:** mouse, lymphatic drainage, lymph flow, collector lymph node, lymphatic mapping, intranodal lympho‐venous shunt

## Abstract

Understanding the lymphatic network is crucial for immunological research. Currently, a complete map of lymphatic drainage in mice is lacking. We present a detailed lymphatic system flow dynamic of two mouse strains with swollen lymph nodes (LNs), using region‐specific tracer injection and high‐resolution micro‐CT imaging to characterize LN volume, weight, density, and spatial topology. No significant differences were observed in LN localization or numbers by strain or sex. Notably, we identified previously unreported drainage pathways and asymmetries, including distinct right and left lymphatic flows. We also discovered intranodal lympho‐venous shunts in LNs, which facilitate unidirectional fluid transport and prevent interstitial fluid buildup and edema. Our findings suggest that these shunts may play a significant role in the delivery of therapeutics within LNs and highlight the need for further research into lymphatic structure–function relationships. © 2026 The Author(s). *The Journal of Pathology* published by John Wiley & Sons Ltd on behalf of The Pathological Society of Great Britain and Ireland.

## Introduction

Obtaining an accurate anatomical and physiological characterization of the lymphatic system is essential for diagnosing and treating lymph node (LN) metastasis [1, 2, 3]. Due to the physio‐anatomical features of the unidirectional valvular structure, lymphatic vessels typically transport lymph upward without backflow in the absence of active pumping. Numerous preclinical studies have explored sentinel LN biopsy and lymphatic rerouting following lymphadenectomy [4, 5]. Consequently, most investigations have been limited to localized regions − often the forepaw or hindfoot − and have not addressed the entire body lymphatic system architecture.

In the current study, we utilized a recombinant inbred swollen LN mouse model [6] consisting of MXH10/Mo‐*lpr*/*lpr* [6, 7] and MXH51/Mo‐*lpr*/*lpr* [6, 8] strains, which were derived from MRL/MpJJmsSlc‐*lpr*/*lpr* [9, 10, 11] and C3H/HeJJmsSlc‐*lpr*/*lpr* [12, 13] mice. Throughout more than 20 generations of intercrossing, both strains developed systemic LN swelling from 12 to 16 weeks of age. The MXH10/Mo/lpr mouse model has superficial LNs approximately 10 mm in diameter and serves as a suitable model for studying LN metastasis and treatment with chemotherapy, radiotherapy, immune checkpoint inhibitors, and combination therapies. MXH10/Mo/lpr mice are known to possess 22 types of LNs [14, 15, 16]. Therefore, the MXH10/Mo/lpr mouse model was instrumental in proposing the LN‐mediated hematogenous metastasis theory [17], which describes a new route for tumor cell dissemination, starting directly from LNs to the systemic circulation via blood vessels overlying the LN surface. Numerous researchers have reported similar findings [18, 19, 20]. According to the LN‐mediated hematogenous metastasis theory, LNs serve as the initial point for distant metastasis in the early stage of lymph node metastasis. Tumor cells within LNs extravasate into penetrating blood vessels on the LN surface, which regulate intranodal pressure, and subsequently enter the systemic circulation prior to dissemination of tumor cells into the systemic circulation from the primary tumor. Building on the MXH10/Mo/lpr mouse model, we previously elucidated a triangular lymphatic flow [21, 22, 23] among the proper axillary LN (PALN), the accessory axillary LN (AALN), and the subiliac LN (SiLN), laying the groundwork for a novel lymphatic drug delivery system (LDDS) [22, 24, 25, 26, 27, 28, 29]. However, the drainage patterns involving internal LNs, such as profundus or inguinal LNs, have remained poorly defined. In the present study, we systematically mapped whole‐body lymphatic system drainage patterns in two distinct mouse strains using a combination of visible dyes and contrast‐enhanced micro‐computed tomography (CT) and magnetic resonance imaging (MRI), achieving unprecedented clarity while preserving the intact lymphatic system.

## Materials and methods

### Ethical approval

Mouse experiments were approved by the Institutional Animal Care and Use Committee of Tohoku University, in line with ARRIVE guidelines 2.0 [30], and were conducted in accordance with ethical guidelines [31].

### Animals

Inbred recombinant MXH10/Mo/lpr (MXH10/Mo‐*lpr*/*lpr*) [6, 7] and MXH51/Mo/lpr (MXH51/Mo‐*lpr*/*lpr*) [6, 8] mice, aged 12–18 weeks, were used in the study to determine unequivocally the lymphatic drainage pattern of experimental rodents. These mice [6] were first inbred at Tohoku University in 1989 by intercrossing MRL/lpr (MRL/MpJJmsSlc‐*lpr*/*lpr*) [9, 10, 11] and C3H/lpr (C3H/HeJJmsSlc‐*lpr*/*lpr*) mice [12, 13]. MXH10/Mo/lpr and MXH51/Mo/lpr mice have enlarged lymph nodes (LNs) similar to humans without inducing fatal auto‐immune diseases like those found in MRL/lpr and C3H/lpr mice. Mice were bred in specific pathogen‐free conditions and housed with no more than four mice per cage with access to food and water *ad libitum*. Mice were exposed to a 12 h light:12 h dark cycle and the temperature was maintained at a comfortable 24 °C. Mice were humanely euthanized immediately after lymphatic drainage mapping. All experimental procedures were carried out under 2% isoflurane (Pfizer Inc., New York, NY, USA) in oxygen for anesthesia, without administration of a lethal dosage.

### Similarity checks among MXH10/Mo/lpr and MXH51/Mo/lpr mice

We performed an anatomical examination to identify differences and similarities between the MXH10/Mo/lpr and MXH51/Mo/lpr mice. Each organ was weighed when the mice were 16–20 weeks old (*n* = 31 for MXH10/Mo/lpr mice and *n* = 9 for MXH51/Mo/lpr mice). In brief, the mice were positioned supine on the dissecting table under an operating microscope, and a sagittal midline incision was made to expose all internal organs, including the inguinal LNs, after general anesthesia was induced by inhalation of 2% isoflurane in oxygen. All organs were identified, photographed, and collected without damage to their structures or residual tissue. The locations of LNs were confirmed by anatomical nomenclature and photographs. The organs were weighed using containers (Iwasaki Industries Co., Ltd., Yamatokoriyama, Nara Prefecture, Japan) placed inside the chamber of an HR‐200 balance (A&D Instruments Ltd., Abingdon, Oxford, UK), and all measurements were carefully recorded.

### Statistical analyses

Data are presented as the mean ± standard error of the mean (SEM). Differences between groups were evaluated using two‐way ANOVA and Tukey's *post hoc* test using GraphPad Prism 9.4.1 (GraphPad Software Inc., La Jolla, CA, USA). A *p* value less than 0.05 was considered statistically significant.

### Lymphatic flow drainage pattern identification

Two experimental methods were used: (1) naked‐eye observations using different Indian inks or an orange dye; and (2) micro‐CT observations using contrast agents. All terminology and localization of LNs were confirmed by three researchers and three expert head and neck surgeons. Mice were positioned on a dissecting table under an operating microscope. After the mice were anesthetized with 2% isoflurane (Pfizer) in oxygen, laparotomy was performed to visualize vital organs and LNs. Before the injection of tracers, the location of organs, especially the LNs, was confirmed and photographed. Additionally, a 1 ml syringe with a 27 G needle (Terumo Co., Tokyo, Japan) filled with 200 μl of black Indian ink or orange dye. eXIATM160XL (contrast agent, 160 mg I/ml; Binitio Biomedical, Ottawa, ON, Canada) was used as a lymphatic flow tracer. 60 μl of the same tracing solution was injected into the superficial LNs, profundus LNs, the forepaw, and the hindfoot at a bolus rate of 2,400 μl/min [24] to visualize lymphatic flow between LNs and identify downstream LNs connected to the injected LNs.

For naked‐eye observations, tracers were injected into unilateral LNs to identify the lymphatic flow pattern. Two different colored tracers were injected into the bilateral LNs to assess symmetrical versus asymmetrical flow patterns, as well as lymphatic vessel branching on both sides. The LNs were harvested, counted, and weighed, and then hemisected to ensure that the tracer was in the internal side of the possible downstream LNs of the targeted LNs. Naked eye visualization opinions were imaged with a camera pro‐function of an Android smartphone.

For micro‐CT observations, mice received an appropriate volume of the contrast agent into the target LNs to visualize lymphatic flow patterns together with afferent and efferent lymphatic vessels and their brachialis. Micro‐CT imaging was performed using a small animal micro‐CT imaging system (CosmoScan GXII; Rigaku Co., Tokyo, Japan) [32] with FOV72 (90 kV, 88 mA). Whole‐body scans or high‐resolution scans with 0.29 μm slice thickness, were acquired at 1‐min intervals, beginning 5 min after injection.

Resovist (200 μl; iron oxide nanoparticle for MRI contrast agent; Kyowa CritiCare Co., Ltd., Atsugi City, Japan) was injected intranodally into the popliteal LN, upstream of the sciatic LN, or intravenously into the tail vein to determine the fluid exchange origin of the intranodal lympho‐venous shunt and its role. LNs were collected immediately after the injection and 5 min post‐injection, then stained with H&E and Berlin Blue, and double‐immunostained with CD31 or LYVE1 alongside Berlin Blue.

### Inclusion and exclusion criteria

Eligibility criteria included a body weight of 30–40 g for a healthy mouse and accurate injection techniques without tracer leakage.

The main exclusion criteria were tracer leak into the abdomen, which could confound lymphatic drainage patterns; absence of the external iliac or colic LNs; or technical disqualification issues, such as unfocused images or missing data.

### Assessment of lymphatic flow and drainage using tracer parameters

Distilled water (DW) was used as a reference sample (control) to ensure precision and accuracy of the measurement device and method. The viscosity of each ink/dye was measured by SV‐1A (two tuning‐16 fork vibration viscometers; A&D Co., Tokyo, Japan) at room temperature (24.6–25.7 °C) [32]. The osmotic pressure of each ink or dye was measured using a Semi‐Micro Osmometer K‐7400 (KNAUER Wissenschaftliche Geräte GmbH, Berlin, Germany).

### Visualization of the lymphatic vessel between the proper axillary LN (PALN) from the subiliac LN (SiLN) and PALN

A 0.5 mm solution of 5(6)‐carboxyfluorescein (MW 376; excitation 492 nm; emission 517 nm; Sigma‐Aldrich Japan, Tokyo, Japan) was injected into the SiLN to assess the presence of sagittal longitudinal midline blood vessel flux to the PALN, and into the AALN to distinguish blood/lymphatic vessels between the AALN and PALN. A 1 ml syringe connected to a 27 G Surflo needle was filled with the appropriate solution, mounted onto a syringe pump (Legato100; KD Scientific, Inc., Holliston, MA, USA), and injected into the target LNs after surgical exposure. Images were captured using a fluorescence stereomicroscope (M165‐FC; fluorescent filter GFP2; excitation 460–500 nm; emission > 510 nm; Leica, Bensheim, Germany) connected to a high‐speed camera (Cool SNAP HQ2; Photometrics, Tokyo, Japan). Images were processed into MP4 video files using MetaVue software (Molecular Devices Corporation, Downingtown, PA, USA).

### Histopathological analyses

LNs and visceral organs were collected and fixed overnight in 10% formalin, followed by dehydration twice and paraffin embedding (FFPE). FFPE tissues were sectioned at a thickness of 3 μm and stained with H&E, as well as immunohistochemical staining (CD31 and LYVE1) and Berlin Blue staining for iron detection (Resovist). Histopathological evaluation was conducted in a double‐blinded manner by two experienced researchers. Representative images were captured at ×10 and ×20 magnification using an Olympus DP‐23 microscope (Olympus Corporation, Tokyo, Japan).

#### H&E staining

Following dewaxing, rehydrated sections were immersed in hematoxylin (Muto Pure Chemicals Co., Ltd, Tokyo, Japan). Differentiation was performed using 1% hydrochloric acid, followed by bluing in distilled water for 5 min. Sections were immersed in 1% eosin for 10 min for cytoplasmic staining. Subsequently, sections were dehydrated, cleared, and mounted with Permount medium.

#### Berlin Blue staining

FFPE sections underwent deparaffinization and rehydration, and were washed in distilled water three times (5 min per wash). Sections were immersed in Berlin Blue – a mixture of equal parts of 2% potassium ferrocyanide and 1% hydrochloric acid, prepared just before use – for 30 min at room temperature. After a further three washes in distilled water (5 min per wash), the sections were counterstained with nuclear fast red (Kernechtrot) for 5 min, followed by dehydration, clearance, and mounting.

#### Immunohistochemistry staining

FFPE sections underwent deparaffinization and rehydration before being subjected to antigen retrieval using an autoclave with 0.01 m citric acid at pH 6.0, maintained at 120 °C for 5 min. After cooling to room temperature, the slides were washed three times with PBS for 5 min each. Primary antibodies, anti‐CD31 (Abcam Inc., Waltham, MA, USA; ab28364, 1:250) and anti‐LYVE1 (ReliaTech GmbH, Wolfenbüttel, Germany; 103‐PA50AG, 1:250), were incubated overnight at 4 °C. The next day, slides were washed and blocking was achieved using 0.3% peroxidase in methanol at room temperature for 20 min. Subsequently, the slides were washed again, and the secondary antibody [Max‐Po(R), Histofine; Nichirei Corporation, Tokyo, Japan] was added at room temperature for 45 min. Color development was achieved with DAB (Histofine), followed by counterstaining with Berlin Blue or Maya Hematoxylin, followed by dehydration, clearance, and mounting.

## Results

**Figure 1:**
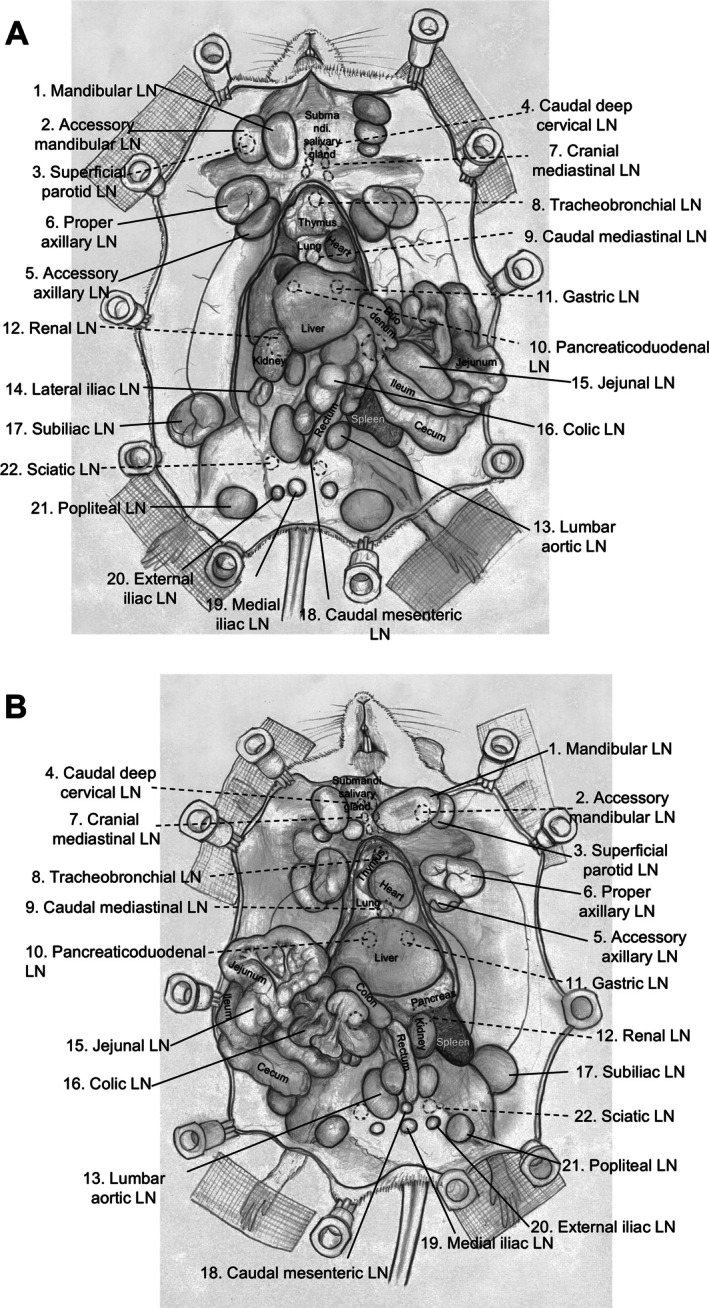
Illustration of swollen lymph node mouse model. (A) Male; (B) female. Anatomical location of mouse organs and 22 types of lymph nodes.

LN topology was performed for the identification and similarity check of MXH10/Mo/lpr and MXH51/Mo/lpr mice (supplementary material, Figure S1A,B). No differences were observed between the numbers, diameters, and localizations of LNs for these strains or for gender (Figure 1A,B and supplementary material, Figure S1Ca,Cb). The long and short axes and weights of the LNs excised from each mouse (*n =* 10) were measured to estimate LN volume and weight per LN (Table 1). The LNs were divided into five regions based on their anatomical locations. The colic LN was challenging to locate in the mouse used for lymphatic topology. Based on Table 1, we summarized LN volume, weight, and numbers per region (Table 2). The average LN density was 1.06 mg/mm^3^, and the number of LNs per g of body weight was 1.34. Average body weights were 34.3 ± 0.7 and 31.9 ± 1.3 g, and the lymphoid organs were 254.2 ± 17.3 and 281.2 ± 18.6 mg for the MXH10/Mo/lpr and MXH51/Mo/lpr mice, respectively (supplementary material, Table S1).

**Table 1:** Median length, diameter, volume, and weight of each lymph node (LN) per region (*n* = 10).

Name of the LN			Long axis (mm)	Short axis (mm)	Volume (mm^3^)	Weight (mg)
Head and neck region lymph nodes						
1	Mandibular LN	R	9.6 ± 0.7	6.0 ± 0.4	184.3 ± 0.1	211.1 ± 50.1
		L	10.4 ± 0.8	7.1 ± 0.8	276.6 ± 0.0	228.5 ± 54.4
2	Accessory mandibular LN	R	4.1 ± 1.1	4.0 ± 1.1	34.6 ± 0.7	60.4 ± 30.0
		L	8.2 ± 1.2	5.8 ± 0.7	144.2 ± 0.3	169.2 ± 29.2
3	Superficial parotid LN	R	6.3 ± 1.3	4.3 ± 1.0	60.6 ± 0.7	223.4 ± 41.5
		L	6.0 ± 1.4	4.7 ± 1.3	70.2 ± 1.2	46.0 ± 33.5
4	Caudal deep cervical LN	R	3.7 ± 0.7	2.5 ± 0.4	12.3 ± 0.2	14.1 ± 4.9
		L	3.0 ± 0.4	2.1 ± 0.2	6.8 ± 0.0	12.5 ± 0.0
Upper limb region lymph nodes						
5	Accessory axillary LN	R	10.7 ± 1.0	7.2 ± 0.4	287.5 ± 0.1	325.3 ± 93.1
		L	9.8 ± 0.7	7.3 ± 0.4	272.4 ± 0.0	199.0 ± 0.0
6	Proper axillary LN	R	10.7 ± 0.6	6.7 ± 0.6	254.1 ± 0.1	192.1 ± 47.9
		L	9.6 ± 0.3	6.6 ± 0.7	221.6 ± 0.1	209.6 ± 62.0
Chest region lymph nodes						
7	Cranial mediastinal LN	R	4.5 ± 0.8	3.4 ± 0.7	26.9 ± 0.2	137.6 ± 94.0
		L	3.1 ± 0.2	2.0 ± 0.1	6.6 ± 0.0	1 ± 0.0
8	Tracheobronchial LN		4.7 ± 0.4	3.6 ± 0.5	31.0 ± 0.0	16.9 ± 5.1
9	Caudal mediastinal LN		2.3 ± 0.0	2.8 ± 0.0	9.4 ± 0.0	1.1 ± 0.0
Abdominal region lymph nodes						
10	Pancreaticoduodenal LN		2.3 ± 0.7	1.6 ± 0.3	3.3 ± 0.0	4.0 ± 1.2
11	Gastric LN		3.5 ± 0.6	2.2 ± 0.6	8.8 ± 0.1	12.7 ± 4.9
12	Renal LN	R	6.8 ± 1.6	4.3 ± 1.1	65.8 ± 1.0	70.4 ± 31.9
		L	6.5 ± 1.1	3.9 ± 0.8	52.2 ± 0.4	39.4 ± 17.1
13	Lumbar aortic LN	R	5.7 ± 0.8	4.8 ± 0.3	69.4 ± 0.0	61.3 ± 19.6
		L	4.6 ± 0.5	3.7 ± 0.1	32.6 ± 0.0	27.9 ± 2.8
14	Lateral iliac LN	R	9.8 ± 0.6	6.1 ± 0.7	189.5 ± 0.1	190.3 ± 75.6
		L	7.5 ± 0.0	5.7 ± 0.0	128.1 ± 0.0	71.8 ± 0.0
15	Jejunal LN		4.5 ± 0.6	3.2 ± 0.2	23.7 ± 0.0	15.0 ± 5.2
						
16	Subiliac LN	R	10.9 ± 0.8	7.6 ± 0.5	328.0 ± 0.1	272.9 ± 33.6
		L	9.5 ± 1.7	7.1 ± 1.5	253.9 ± 1.9	291.3 ± 98.2
Lower limb region lymph nodes						
17	Caudal mesenteric LN		7.8 ± 1.5	4.9 ± 0.2	96.5 ± 0.0	121.4 ± 46.7
18	Medial iliac LN		3.9 ± 0.0	3.6 ± 1.0	26.0 ± 0.0	13.7 ± 0.5
19	External iliac LN	R	3.2 ± 0.0	2.3 ± 0.0	8.7 ± 0.0	3.6 ± 0.0
		L	4.2 ± 0.0	2.7 ± 0.0	16.3 ± 0.0	8.2 ± 0.0
20	Popliteal LN	R	9.2 ± 0.8	8.3 ± 0.7	328.8 ± 0.2	318.3 ± 86.8
		L	8.9 ± 0.4	7.7 ± 0.2	273.3 ± 0.0	228.1 ± 33.1
21	Sciatic LN	R	7.7 ± 2.2	5.6 ± 1.0	126.5 ± 1.3	235.9 ± 139.6
		L	9.3 ± 2.6	7.4 ± 2.1	263.5 ± 5.8	404.9 ± 266.5

**Table 2:** Number of LNs per region and LNs/g (*n* = 10).

LN region	LN volume summary (mm^3^)	Weight summary of the LNs (mg)	LN density (mg/mm^3^)	LN number/mouse	Body weight (g)	Number of LNs/g
Head and neck region LNs	789.6 ± 3.2	965.2 ± 243.6	1.06	14.8	40	1.34
Upper limb region LNs	1,035.6 ± 0.3	926 ± 203		4		
Chest region LNs	73.9 ± 0.2	156.6 ± 99.1		4.7		
Abdominal region LNs	1,155.3 ± 3.6	1,057.0 ± 290.1		19.6		
Lower limb region LNs	1,139.6 ± 7.3	1,334.1 ± 573.2		10.5		
Total	4,194 ± 14.6	4,438.9 ± 1,409		53.6		

**Figure 2:**
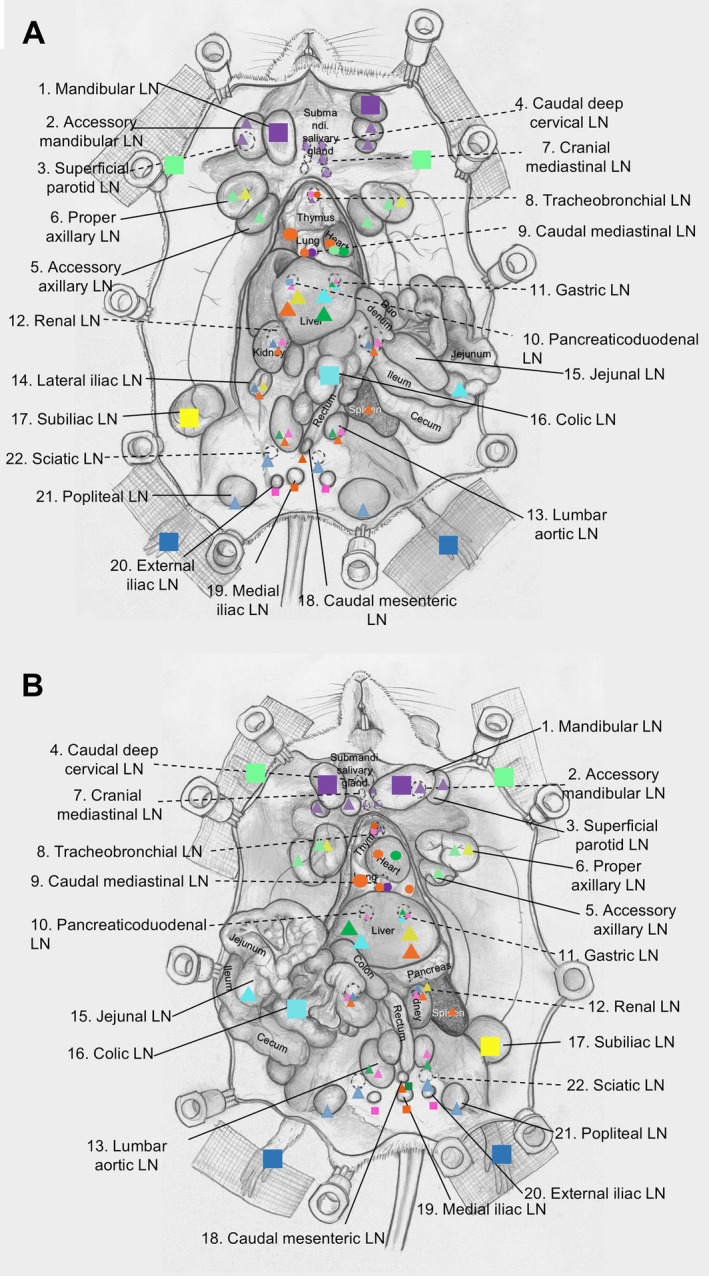
Lymph flow pattern of the entire mouse body. (A) Male; (B) female. ■, Ink/dye injection site; ▲, downstream LNs of the injection site; ●, endpoint of the flow; dashed lines, lymphatic vessels between LNs.

Average LN volumes were 98.7 ± 0.4 mm^3^ for the head and neck region, 18.5 ± 0.1 mm^3^ for the chest region, 258.9 ± 0.1 mm^3^ for the upper limb region, 104.0 ± 0.3 mm^3^ for the abdominal region, 142.5 ± 0.9 mm^3^ for the lower limb region, and, overall, 119.8 ± 0.4 mm^3^ for the entire body (Table 2). The average long axis of lymph nodes (LNs) was approximately 10 mm in the chest region and 6–8 mm in other regions, and the overall average was 7 mm. The average short axis of LNs was 6.9 mm in the chest region and 4–6 mm in other regions, and the overall average was 5 mm. The maximum average weight of LNs was 231.5 ± 50.8 mg for the upper limb region, while the minimum was 39.2 ± 24.8 mg in the chest region. The greatest number of LNs was located in the abdominal region, followed by the head and neck region, the lower limb region, and similar numbers of LNs were found in the upper limb and chest regions (Table 3). Figure 2 illustrates the entire lymphatic drainage pattern determined from a specific injection site.

**Table 3:** Average lymph node volume and axis length by region (*n* = 4).

LN region	LNs*	Average LN volume (mm^3^)	Average long axis of LN (mm)	Average short axis of LN (mm)	Average weight of the LNs (mg)	LN number /mouse
Head and neck	1, 2, 3, 4	98.7 ± 0.4	8.1 ± 0.8	5.5 ± 0.4	120.7 ± 30.5	14.8
Chest	10, 11, 12	18.5 ± 0.1	10.0 ± 0.4	6.9 ± 0.3	39.2 ± 24.8	4.7
Upper limb	5, 6	258.9 ± 0.1	6.2 ± 1.0	4.5 ± 0.8	231.5 ± 50.8	4
Abdominal	7, 13, 14, 15, 16, 18, 19, 20	104.0 ± 0.3	7.5 ± 0.7	5.7 ± 0.6	96.1 ± 26.4	19.6
Lower limb	8, 9, 17, 21, 22	142.5 ± 0.9	6.9 ± 0.9	5.3 ± 0.7	166.8 ± 71.4	10.5
Entire body	All	119.8 ± 0.4	7.0 ± 0.3	5.0 ± 0.2	126.8 ± 40.3	53.6

* Numbers 1–22 correspond to those listed in Table 1.

### Lymphatic drainage of the head and neck region LNs

Two different lymphatic drainage patterns were found from the right or left mandibular LN (supplementary material, Figure S2A–C). The most common drainage pattern was from the right (R.) mandibular LN to the R. accessary mandibular LN, then to the R. superficial parotid LN, and subsequently to the R. caudal deep cervical LN (supplementary material, Figure S2A); clear visualization of the connecting lymphatic vessels was obtained for all subsequent routes described below. An alternative lymphatic drainage route was observed from the R. mandibular LN flow into the left (L.) accessary mandibular LN and then into the L. superficial parotid LN. Conversely, the L. mandibular LN flowed to the R. accessary mandibular LN and then to the R. superficial parotid LN (supplementary material, Figure S2B).

The lymphatic drainage pattern from the R. accessary mandibular LN proceeded to the L. accessory mandibular LN, R. and L. superficial LNs, and subsequently to the R. and L. caudal deep cervical LNs (supplementary material, Figure S2C). The drainage route from the L. accessory mandibular LN was to the R. and L. superficial parotid LNs, then to the R. and L. caudal deep cervical LNs, and finally to the L. cranial mediastinal LN (supplementary material, Figure S2C,D).

Drainage from the R. superficial parotid LN proceeded to the R. caudal deep cervical LN and then to the R. caudal deep cervical LN (supplementary material, Figure S2E,F). Lymphatic drainage patterns were also detected from the L. mandibular LN to the L. accessory mandibular LN, then to the L. superficial LN, followed by the L. caudal deep cervical LN, the L. caudal deep cervical LN, the L. cranial mediastinal LN, the tracheobronchial LN, and finally the caudal mediastinal LN.

### Lymphatic drainage of upper limb region LNs

Tracer injected into the R. forepaw flowed to the R. AALN and then to the R. PALN (supplementary material, Figure S3A), whereas tracer injected into the L. forepaw flowed to the L. AALN, then to the L. PALN, and finally to the lung (supplementary material, Figure S3A). Tracer injected into the R. AALN flowed to the R. PALN (supplementary material, Figure S3B) and was consistent with the pattern observed after R. forepaw injection. Tracer injected into the L. AALN flowed to the L. PALN and then to the lung (supplementary material, Figure S3B), mirroring the pattern observed after L. forepaw injection. Efferent lymphatic vessels from the PALN to the thoracic cavity were only found on the right side. Fluorophores injected into the R. AALN entered the PALN and exited via the efferent lymphatic vessels of the R. PALN before joining the vena subclavia and systemic circulation (supplementary material, Figure S3C).

### Lymphatic drainage of abdominal region LNs

Tracer injected into the gastric LN flowed to the pancreaticoduodenal LN and jejunal LNs (supplementary material, Figure S4A). Tracer injected into the pancreaticoduodenal LN flowed to the liver, jejunal LNs, and spleen, and then upwards to the cardiorespiratory system in clearly defined lymphatic vessels (supplementary material, Figure S4B). Colic LN injected tracer flowed to the pancreaticoduodenal LN, gastric LN, and jejunal LNs, and then in an upwards direction to the liver and cardiorespiratory system (supplementary material, Figure S4C). In some mice, lymphatic flow from the colic LN did not extend further than to the jejunal LNs (supplementary material, Figure S4D). Tracer injected into the lumbar aortic LN flowed to the same side of the, then upwards to the cardiorespiratory system (supplementary material, Figure S4E).

In some cases, tracer injected into the R. lumbar aortic LN flowed to the R. renal LN, liver, and lung, and then to the tracheobronchial LN (supplementary material, Figure S4F). In rare cases, tracer injected into the R. lumbar aortic LN flowed to the R. renal LN and spleen, and then to the tracheobronchial LN (supplementary material, Figure S4G). Tracer injected into the R. lateral iliac LN flowed to the R. renal LN, pancreaticoduodenal LN, gastric LN, and liver, and was then routed upwards to the cardiorespiratory system (supplementary material, Figure S4H). Tracer injected into the L. lateral iliac LN flowed to the L. lumbar aortic LN and L. renal LN, and then in an upwards direction to the cardiorespiratory system (supplementary material, Figure S4I). It is noteworthy that in several cases the tracer injected into the R. lateral iliac LN flowed to the same side of the renal LN and the ipsilateral side of the renal LN and gastric LN (supplementary material, Figure S4J). When different colored tracers were injected into both sides of the lateral iliac LN (20%), similar flow patterns were observed for individual injections (supplementary material, Figure S4K,L).

R. SiLN injected tracer flowed to the R. PALN through the lymphatic vessel joining the SiLN and PALN, and the blood vessel between the SiLN and PALN. Tracer flowed from the vena thoracoepigastric to the vena cava inferior (liver) through the lymphatic vessel to the R. lateral iliac LN via the R. vena superficial caudal epigastric to the L. vena popliteal (supplementary material, Figure S4M,N). L. SiLN injected tracer flowed to the L. PALN through the lymphatic vessel connecting the SiLN, PALN, and L. renal LN. Interestingly, a novel blood vessel (supplementary material, Figure S4Md,Nd) was observed running between both sides of the SiLN to the PALN, following a longitudinal path along the midline of the frontal axis (supplementary material, Extended Video S1A). To ensure visualization of this blood vessel, a midline longitudinal incision should be avoided.

### Lymphatic drainage of lower limb region LNs

Caudal mesenteric LN injected tracer flowed to both sides of the lateral iliac LN, the L. lumbar aortic LN, both sides of the renal LN, the liver, and then to the gastric LN (supplementary material, Figure S5A). In some cases, tracers were observed to enter the L. renal LN after flowing to both sides of the lateral iliac LN (supplementary material, Figure S5B). Caudal mesenteric LN injected tracer flow was sometimes limited to the L. lateral iliac LN (supplementary material, Figure S5C and Extended Video S1B). In rare cases, tracers from the caudal mesenteric LN did not enter the L. lateral iliac LN but rather directly flowed into the L. renal LN and gastric LN (supplementary material, Figure S5D). Medial iliac injected tracers flowed into the R. lateral iliac and renal LNs and spleen, and were then routed upwards to the heart (supplementary material, Figure S5E).

In some cases, tracer injected into the medial iliac LN flowed into the caudal mesenteric LN, both sides of the lateral iliac LNs, renal LNs, the liver, lung, and caudal mediastinal LN (supplementary material, Figure S5F). Tracer injected into the R. external iliac LN flowed to the R. lumbar aortic LN, then to both sides of the renal LN (supplementary material, Figure S5G). L. external iliac LN injected tracers flowed to the L. lumbar aortic LN, both sides of the renal LNs, the pancreaticoduodenal LN, and then to the gastric LN (supplementary material, Figure S5G). Individually injected tracers flowed from the L. external iliac LN to the L. lumbar aortic LN, the L. renal LN, and then to the tracheobronchial LN (supplementary material, Figure S5H). It was found that the external iliac LN was gender‐specific, and the presence ratio of the external LN was 11:1 (male:female) for lymphatic drainage flow from the R. or L. sciatic LN, determined using two different colored tracers. The lymphatic flow was symmetrical on both sides (supplementary material, Figure S5I), first entering the injection site, i.e. the lateral iliac LN, and then the renal LN. There was clear visualization of the lymphatic vessels connecting the lateral iliac LN and the renal LN. Two different colored tracers were injected into the R. or L. popliteal LN, respectively. Lymphatic drainage flow was found to occur at the injection site, to the sciatic LNs, and followed the same routes as the sciatic LN drainage pattern (supplementary material, Figure S5J). Tracers injected into both sides of the hindfoot flowed to the injection site popliteal LNs, and the routes followed were essentially the same as the popliteal LN drainage pattern.

In addition, lymphatic drainage patterns were validated using *in vivo* micro‐CT imaging to detect invisible lymphatic vessel bifurcations or unexpected novel routes. The head and neck region LNs’ lymphatic drainage pattern was similar to the typical flow pattern revealed by ink/dye. In brief, contrast agents injected into the mandibular LN demonstrated lymphatic flow to the accessory mandibular LN, superficial parotid LN, caudal deep cervical LN of the injection site, and lymphatic vessels between these LNs as revealed by micro‐CT imaging (supplementary material, Figure S6A). The lymphatic drainage pattern of the upper limb region LNs was the same as that revealed using the ink/dye flow technique. In addition, contrast agent flow was observed to occur from the forepaw to the accessary axillary LN (supplementary material, Figure S6A,B), and from the AALN to the PALN (supplementary material, Figure S6C).

Contrast agent injection into profundus LNs and observation of its subsequent flow was a challenging task; LNs were minimized to prevent confusion in the patterns caused by leakage of contrast agent in the abdominal cavity. The possible injection sites within the profundus LNs were into the colic LN, lateral iliac LN, and inguinal SiLN. For the lower limb region, the caudal mesenteric LN and external iliac LN were imaged from the profundus LNs, and all inguinal LNs were imaged using micro‐CT. The lymphatic drainage pattern of the colic LN revealed by micro‐CT (supplementary material, Figure S7A) and contrast agent flow was subsequently detected in the jejunal LNs, L. renal LN, and gastric LNs (same as supplementary material, Figure S4C), before being routed upwards to the cardiorespiratory system. Contrast agents injected into the lateral iliac LN drained to the lumbar aortic LNs and renal LNs, and then upwards to the cardiorespiratory system, consistent with the pattern shown in supplementary material, Figure S4H,I. Lymphatic drainage from the SiLN (supplementary material, Figure S7C) followed a similar pattern to that observed from the SiLN to the PALN via the lymphatic vessels, as shown in supplementary material, Figure S4M,N.

Contrast agent injected into the caudal mesenteric LN (supplementary material, Figure S8) followed a similar trajectory to that shown in supplementary material, Figure S5A. Flow was observed on both sides of the lateral iliac LN (supplementary material, Figure S8A). Contrast agent injected into the L. external iliac LN flowed to the L. lumbar aortic LN and then to the L. renal LN (supplementary material, Figure S8B). Following injection into the sciatic LN, flow was observed at the injection site of the lateral iliac LN and in the renal LN, with more marked flow on the right side than on the left side, as revealed by micro‐CT (supplementary material, Figure S8C). The lymphatic drainage pattern from the popliteal LN evaluated using micro‐CT (supplementary material, Figure S8D) matched the ink/dye flow pattern shown in supplementary material, Figure S5J. Interestingly, two efferent lymphatic vessels of the popliteal LN (both sides) were found; one routed to the sciatic LN and the other to the lateral iliac LN. When contrast agent was injected into the R. hindfoot, flow was directed into the medial iliac LN (supplementary material, Figure S8E), which is different to the ink/dye results shown in supplementary material, Figure S5K. Conversely, the contrast agent injected into the L. hindfoot flowed to the medial iliac LN and L. popliteal LN (supplementary material, Figure S8E), yielding the same result as in supplementary material, Figure S5K. It is important to note that micro‐CT provided more accurate results than those from the naked‐eye investigations. Notably, tracer flow patterns in the lymphatic system did not differ regardless of the tracer osmotic pressure and viscosity (supplementary material, Figure S1D).

### Lymph–blood vessel barrier inside the LNs

**Figure 3:**
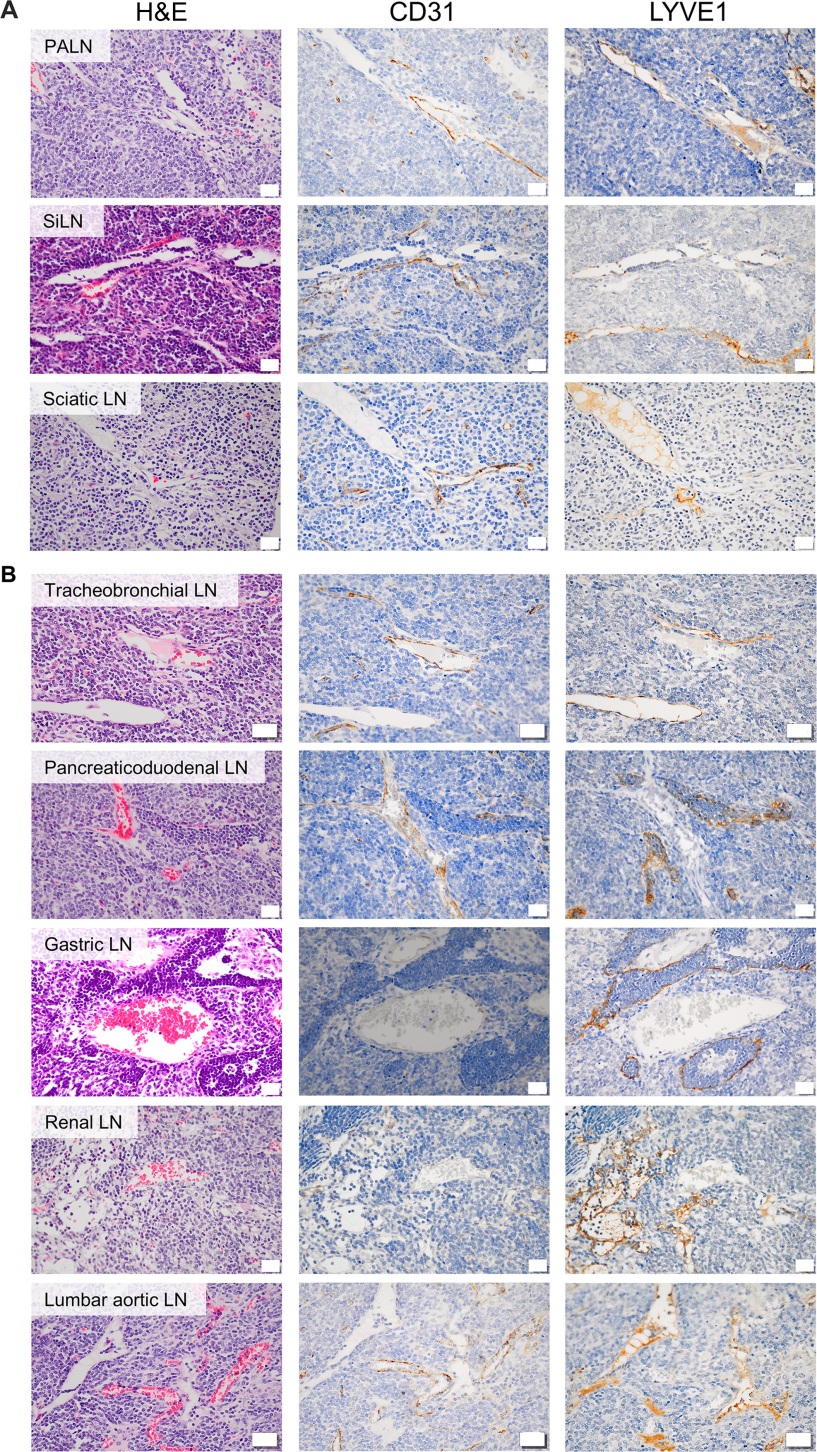
Histological characterization of superficial and profundus lymph nodes with lympho‐venous shunts. (A) Superficial LNs; (B) profundus LNs with lympho‐venous shunt. LNs were collected from robust MXH10/Mo/lpr and MXH51/Mo/lpr mice. LNs were stained with H&E, CD31, and LVYE1. Scale bar, 20 μm.

**Figure 4:**
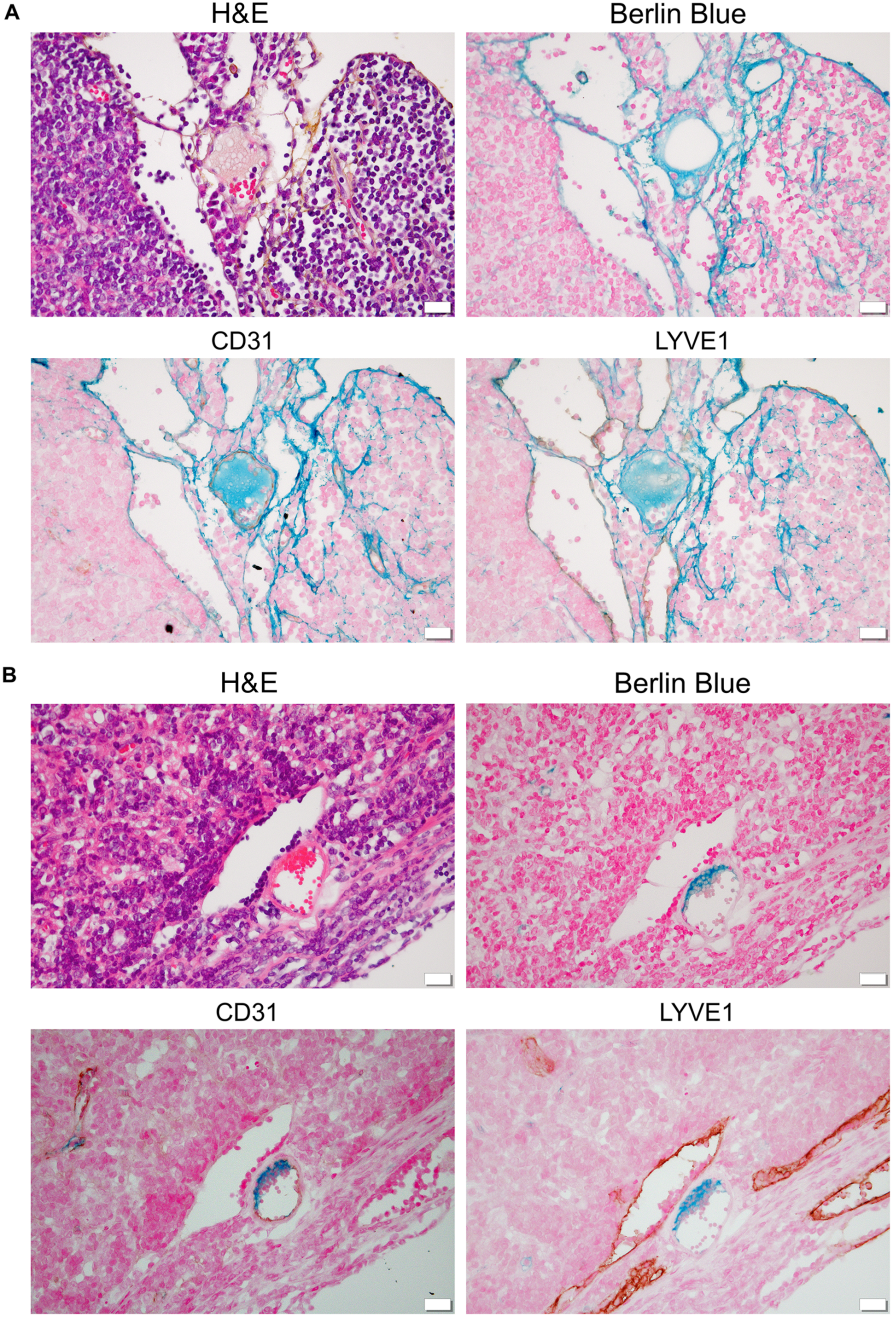
Tracing of intranodally injected Resovist to assess the presence of an intranodal lympho‐venous shunt. Resovist was injected into the popliteal lymph node (LN), upstream of the sciatic LN ((A) Animal 1; (B) Animal 2), and tissues were collected immediately or 5 min post‐injection. Sciatic LN was stained with H&E, Berlin Blue, and double staining with CD31 or LYVE1 to confirm the localization of Resovist.

During our investigation of the lymph and drainage patterns, we observed the co‐existence of lymph sinus and blood vessels in 9 out of 22 LN types (Figures 3 and 4), referred to as the intranodal lympho‐venous shunt. This shunt was present in superficial LNs (Figure 3A) of the PALN, SiLN, and sciatic LN; profundus LNs (Figure 3B), tracheobronchial LN, pancreatoduodenal LN, gastric LN, renal LN, and the lumbar aortic LN. To investigate the origin of fluid exchange through the intranodal lympho‐venous shunt, Resovist was injected into the sciatic LN (downstream LN of the popliteal LN). Resovist was observed in the lymph sinus and the intranodal lympho‐venous shunt of the popliteal LN (upstream of the sciatic LN) injected group, where it was stained blue using Berlin Blue staining regardless of the sampling time. This was confirmed by double staining with CD31 or LYVE1 alongside Berlin Blue (Figure 4A). These findings indicate that Resovist, injected intranodally into the popliteal LN, flowed into the sciatic LN via the lymphatic vessel and subsequently entered the blood vessel within the sciatic LN through the intranodal lympho‐venous shunt. However, in the intravenous injection group, Resovist was detected in blood vessels of the sciatic LN, not in the lymph sinus and the intranodal lympho‐venous shunt (Figure 4B).

**Figure 5:**
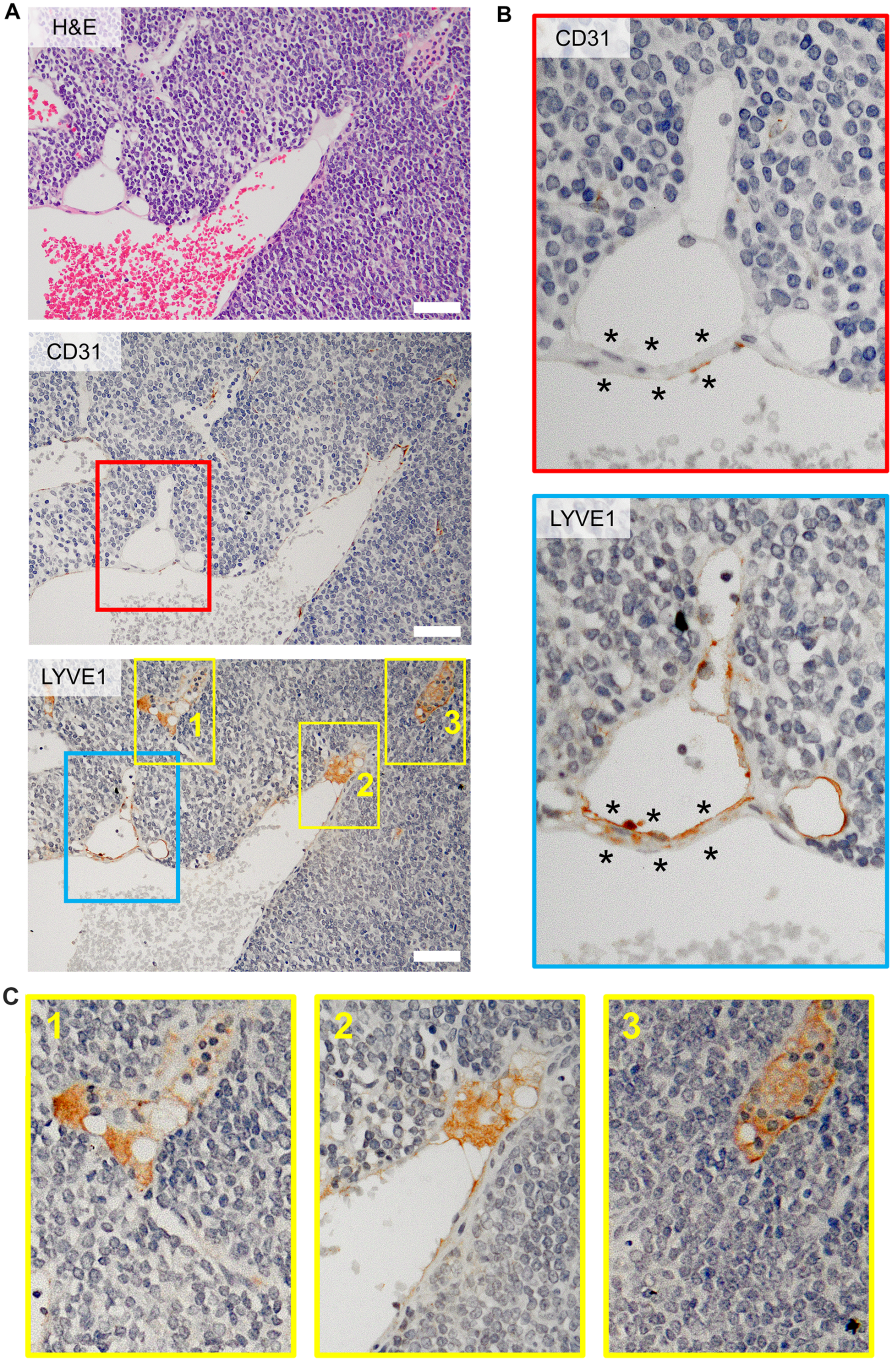
Histological identification of intranodal lympho‐venous shunts in lumbar aortic lymph nodes. (A) Lumbar aortic LN (LN with intranodal lympho‐venous shunt) stained with H&E and CD31 and LYVE1 staining. Scale bar, 50 μm. Red box, area of CD31 staining magnified in B; blue box, area of LYVE1 staining magnified in B; yellow boxes (1–3), lymph fluid with LVYE1‐bound hyaluronic acid magnified in C. (B) Magnified view of CD31 and LYVE1 staining of lumbar aortic LN. Asterisks indicate intranodal lympho‐venous shunts. (C) Magnified views of LYVE1‐bound hyaluronic acid in LYVE1 staining of lumbar aortic LN.

Intranodal lympho‐venous shunt positive lumbar aortic LN was stained with H&E, CD31, and LYVE1 (Figure 5A; magnified view of CD31 and LVYE1 in Figure 5B). In sections of the lumbar aortic LN stained with LYVE1, we observed LYVE1‐bound hyaluronic acid draining from the lymph sinus (Figures 5A(1–3),C and 3B) into the blood vessels (CD31‐positive staining), occurring through the intranodal lympho‐venous shunt (Figure 5A,B). These results confirmed that lymph flow or fluid exchange is unidirectional, proceeding from the lymph sinus to the blood vessels through the intranodal lympho‐venous shunt, rather than from the blood vessels to the lymph sinus.

## Discussion

Previously, studying lymphatic drainage in a conventional mouse model was challenging, particularly for the identification of LNs [15, 16]. In the present study, we confirmed no differences in organ and body weights between the MXH10/Mo/lpr and MXH51/Mo/lpr strains or in the number of LNs (supplementary material, Figure S1C,D and supplementary material, Table S1). Documentation of LN volume and weight represents a novel aspect of this study (Tables 1 and 2). In clinical practice, lymphoscintigraphy of the head and neck region and abdominal area is performed only if patients give formal informed participation consent. Furthermore, the regions that can be studied relatively easily are limited, i.e. maxilla, mandible, and the head and neck area. Surgeons have applied this technique during surgery to improve real‐time assessment of sentinel LNs [33, 34, 35, 36]. However, previous studies were limited by the low numbers of participants and a narrow focus only on the surgical area with limited visibility range and short‐lived, rapidly decaying, and shallow‐penetrating signals, preventing sustained or deep lymphatic visualization [1, 37, 38]. The LN‐swollen mouse model enabled detailed investigation of lymphatic flow patterns within a practical time frame, providing a larger surgical window in the incision area and minimal pain to the animal. No differences in lymphatic flow or fluid dynamics were observed from the SiLN to the PALN in MXH10/Mo/lpr mice compared with C57BL/6J, BALB/cAJcl, and NOD/ShiJic‐scidJcl mice [17]. We anticipate that similar findings would be observed in other wild‐type mouse strains.

The first novel finding was that the mandibular LN acts as a collector LN for the head and neck region; upstream of the axillary mandibular LN, superficial parotid LN and lymphatic vessel anastomosis were found between these LNs (Figure 2).

For the upper limb region, AALN is the collector LN and collecting lymphatic vessels arise from the mouse forepaw; lymph from the left side runs directly into the lung and not from the right side of the upper limb. The PALN is downstream of the AALN and SiLN, with both LNs being collector LNs in their locales. Most interestingly, we observed lymphatic and thoracic ducts in our mouse models similar to those found in humans. Ink/dye injected into the right forepaw was routed to the L. AALN, flowed into the L. PALN, and then into the vena subclavia (Figures 2 and 3 and supplementary material, Figure S3). These findings indicate that the lymphatic duct drains from the left upper limb and merges with the left vena subclavia, not in the head and neck region, and the rest of the lymphatic vessels drain into the thoracic duct.

For abdominal region LNs, lymph flows intra‐abdominally downwards and then upwards; anastomosis between the right and left sides has been found in the lateral iliac LN and lumbar aortic LNs. In brief, lymphatic flow is routed from the SiLN to the lateral iliac LN, lumbar aortic LN, renal LN, pancreaticoduodenal LN, gastric LN, liver, spleen, and then to the thoracic cavity. We believe that a lymphatic vessel shunt exists for abdominal LNs that directs lymph flow to the liver. Surprisingly, we found a new blood vessel connection from the SiLN to the PALN; thus, moieties from the SiLN to the PALN migrate through lymphatic and blood vessels.

**Figure 6:**
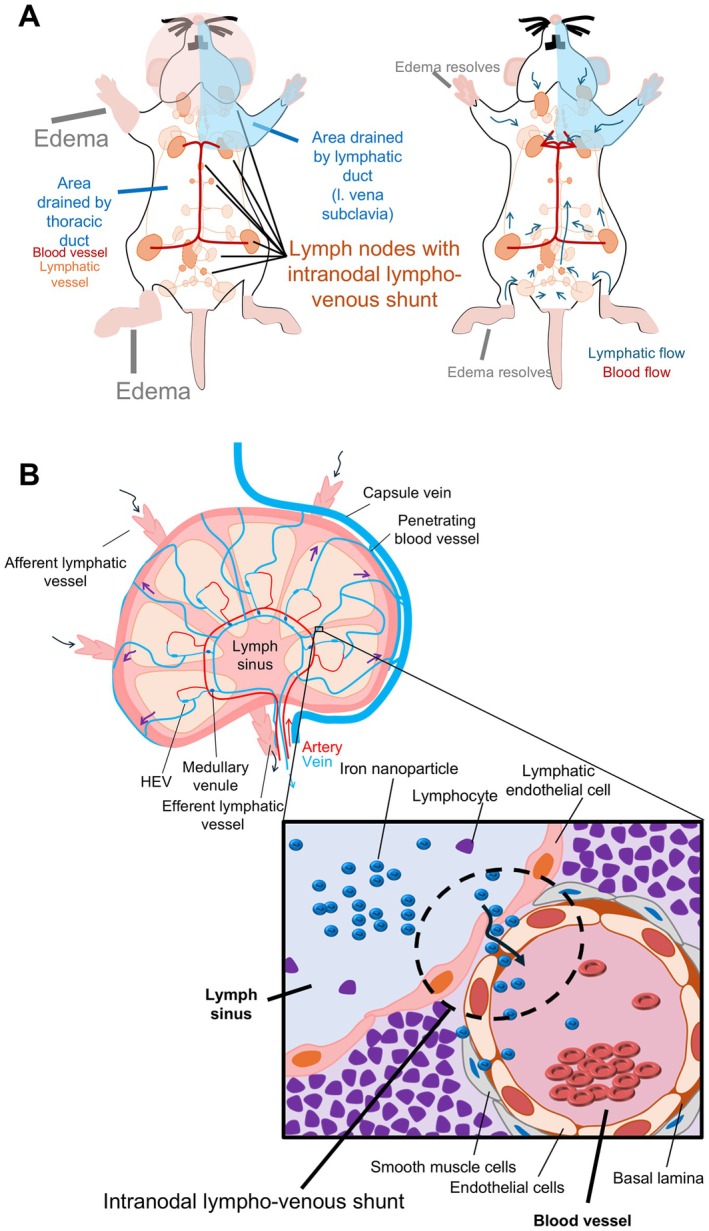
The lymphatic system's topology. (A) Illustration summarizing a schematic that integrates all the results, depicting the lymphatic and thoracic ducts, lymph flow, and LNs featuring an intranodal lympho‐venous shunt, including the concept of the intranodal lympho‐venous shunt role in edema resolution. (B) Illustration of an LN's structure including penetrating blood vessels, the intranodal shunt, high endothelial venule (HEV), and associated fluid dynamics.

For the lower limb region, the initial or collector LN is the popliteal LN. Lymph flows to the sciatic LN, external iliac LN, medial iliac, caudal mesenteric LN, and lateral iliac LN, and then uses the same route as the abdominal region LNs. All these routes were confirmed using micro‐CT imaging at high resolution. Some results may differ due to the physical parameters of the tracers, such as their osmotic pressure and viscosity (supplementary material, Figure S1D). Contrast agent flows more readily to downstream LNs than does dye or ink. We observed sexual dimorphism in the presence of the external iliac LN, consistent with clinical findings [39]. Another major finding was the presence of two efferent lymphatic vessels from the popliteal LN, one routed to the sciatic LN and the other to the lateral iliac LN. Therefore, the lateral iliac LN is downstream of the sciatic and popliteal LNs. All lymphatic flow patterns described above were symmetrically paired in the abdominal and lower limb LNs. Our summary of the evidence indicates that although mice possess thoracic and lymphatic ducts similar to humans, the anatomical locations of these ducts are positioned contralaterally in mice (Figure 6A). An intranodal lympho‐venous shunt was primarily observed in the lymph nodes near the subclavian vein and inferior vena cava (Figures 3, 4, 5). The intranodal lympho‐venous shunt in the PALN facilitates excess lymph fluid flow from the forepaw and AALN into the vena subclavia. Intranodal lympho‐venous shunts in the SiLN, lumbar aortic LN, and sciatic LN drain excess fluid from the hindfoot to the inferior vena cava.

We found evidence of LYVE1‐bound hyaluronic acid [40, 41, 42] as a component of lymph fluid draining from the lymph sinus to blood vessels through an intranodal lympho‐venous shunt (Figure 5). LYVE1, recognized for its role in hyaluronan metabolism, may function as a transporter for hyaluronan. Lymph nodes with intranodal lympho‐venous shunts are therefore thought to play an important role in regulating lymph fluid balance, preventing edema in the forepaw and hindfoot, and potentially reducing swelling in both regions (Figure 6A). In contrast, LNs located near the small and large intestines do not exhibit intranodal lympho‐venous shunts. Taken together, these findings suggest that LNs regulate blood and lymph flow to the organs based on their anatomical location.

Gaining insight into lymphatic flow and drainage patterns will broaden the applications of the lymphatic drug delivery system (LDDS) for the treatment of cancer metastasis in upcoming clinical trials, including an ongoing phase 1 clinical trial in Japan for head and neck cancer using docetaxel [43] (https://jrct.mhlw.go.jp/en-latest-detail/jRCTs021230040) and ipilimumab [44] (https://jrct.mhlw.go.jp/latest-detail/jRCTs021250037). The LDDS delivers chemotherapeutic agents and immune checkpoint inhibitors that inhibit tumor growth and metastasis in LNs, as demonstrated in preclinical studies using superficial LNs. In clinical use, therapeutic agents will be injected under ultrasound guidance, and the main criterion for LNs eligible for injection is a LN size greater than 5 mm, regardless of tumor cell involvement. In our current study, the number of LNs with a long axis of ≤ 5 mm was 46 (39.3%), while the number with a long axis of > 5 mm was 71 (60.7%) throughout the entire bodies of four mice. The number of LNs with a short axis of ≤ 5 mm was 59 (50.4%), while the number with a long axis of > 5 mm was 57 (49.6%) throughout the entire bodies of four mice. We believe that understanding lymph and drainage patterns of the lymphatic system (Figures 2 and 6A) will provide insights into new challenges for using profundus LNs in broad cancer treatment and prevention. In addition, intranodal lympho‐venous shunts inside the LNs (Figures 3, 4, 5 and 6B) may enhance the therapeutic efficacy of the LDDS for LN and distant metastasis, and visceral cancers. Therefore, LNs containing intranodal lympho‐venous shunts and penetrating blood vessels may contribute to edema resolution and prevent its formation (Figure 6A,B), thereby playing an important role in regulating lymph fluid balance.

We understand that the primary function of the intranodular lympho‐venous shunt appears to be the transport and efflux of lymph fluid directly from the lymph sinus to co‐localized blood vessels. In contrast, high endothelial venules (HEVs) facilitate the movement and influx of lymphocytes from the bloodstream into the lymph node parenchyma. Therefore, the function of the intranodal lympho‐venous shunt is the opposite to that of HEVs. This opposing function suggests that LNs not only recruit immune cells but also actively regulate systemic fluid and immune mediator distribution. Intranodal lympho‐venous shunts therefore provide an alternative pathway for draining lymph components such as macromolecules, immune mediators, and potentially cells into the systemic circulation, while avoiding the conventional lymphatic drainage through efferent lymphatics and the thoracic duct. These findings suggest that intranodal lympho‐venous shunts play a more active and integrated role in maintaining fluid balance and regulating the immune system, possibly influencing both antigen distribution and systemic immune reactions. Future studies are underway to clarify the developmental origin and regulatory mechanisms of intranodal lympho‐venous shunts. Understanding these processes may further elucidate their roles in lymphatic drug delivery, metastatic spread, immune modulation, and edema resolution.

The study has several limitations. First, it was designated as a cross‐sectional study, and lymphatic flow under abnormal circumstances was not examined. All findings were based on photographic documentation and histological evaluations. Finally, the lymphatic flow patterns of the mouse system are likely to differ from those of other animal species.

In summary, the lymphatic drainage patterns described will enable more detailed investigations of LN metastasis that more closely replicate clinical conditions in humans. The implementation of new experimental protocols is expected to improve our understanding of metastatic LNs, including their diagnosis and treatment. Elucidation of lymphatic drainage patterns may also facilitate the generation of metastatic LNs or distant organs through lymphatic seeding. We identified lymphatic vessels connecting adjacent lymph nodes, crossing the midline to enter contralateral downstream LNs, and forming anastomotic networks that converged on multiple first‐tier LNs. Although these phenomena have not yet been studied in humans, lymphatic drainage patterns present a new approach for studying cancer metastasis. Additionally, intranodal lympho‐venous shunts may represent a novel approach for preventing and treating edema by regulating fluid exchange and transporting molecules during cancer therapy.

## Author contributions statement

TK and AS designed the experiments. AS and MR carried out the tracer research experiments, and AS carried out the intranodal lympho‐venous shunt experiment. AN assisted with the micro‐CT imaging. AS, MR and MS analyzed the data and agreed on the locations and nomenclature naming of the LNs. MS and AS evaluated the histological analyses. AS summarized data, made illustrations, and wrote the manuscript. All authors approved the definitive version submitted to the journal for publication and agreed to be responsible for all aspects of the research.

## Supporting information


**Figure S1.** Characteristics of the study animals and their LN localizations
**Figure S2.** The lymphatic flow patterns of head and neck region LNs
**Figure S3.** The lymphatic flow patterns of upper limb region LNs
**Figure S4.** The lymphatic flow patterns of abdominal region LNs
**Figure S5.** The lymphatic flow patterns of lower limb region LNs
**Figure S6.** Lymphatic tracing of head and neck region and upper limb region LNs (CT)
**Figure S7.** Lymphatic tracing of abdominal region LNs (CT)
**Figure S8.** Lymphatic tracing of lower limb region LNs (CT)
**Table S1.** Body weight and organ weights of MXH10/Mo/lpr (*n* = 31) and MXH51/Mo/lpr (*n* = 9) mice

**Video S1.** Lymphatic flow imaging (provided as a separate MP4 file)

## Data Availability

Data supporting the findings of the study are available from the corresponding author upon reasonable request.
