# Supplementary material for: Lymphatic topology reveals a novel intranodal lympho‐venous shunt
Source: J Pathol. 2026 Feb 4;268(4):477–92. doi: 10.1002/path.70032 (PMC12984476; doi:10.1002/path.70032)
Supplement: Supplementary file 1 — Figure S1. Characteristics of the study animals and their LN localizations Figure S2. The lymphatic flow patterns of head and neck region LNs Figure S3. The lymphatic flow patterns of upper limb region LNs Figure S4. The lymphatic flow patterns of abdominal region LNs Figure S5. The lymphatic flow patterns of lower limb region LNs Figure S6. Lymphatic tracing of head and neck region and upper limb region LNs (CT) Figure S7. Lymphatic tracing of abdominal region LNs (CT) Figure S8. Lymphatic tracing of lower limb region LNs (CT) Table S1. Body weight and organ weights of MXH10/Mo/lpr (n = 31) and MXH51/Mo/lpr (n = 9) mice [file PATH-268-477-s002.docx]

**Lymphatic topology reveals a novel intranodal lympho-venous shunt**

S Ariunbuyan *et al. J Pathol* <https://doi.org/10.1002/path.70032>

**Supplementary Figures S1–S8**

**Supplementary Table S1**

**Extended Video S1 (provided as a separate MP4 file)**

Reference numbers refer to the main text list.


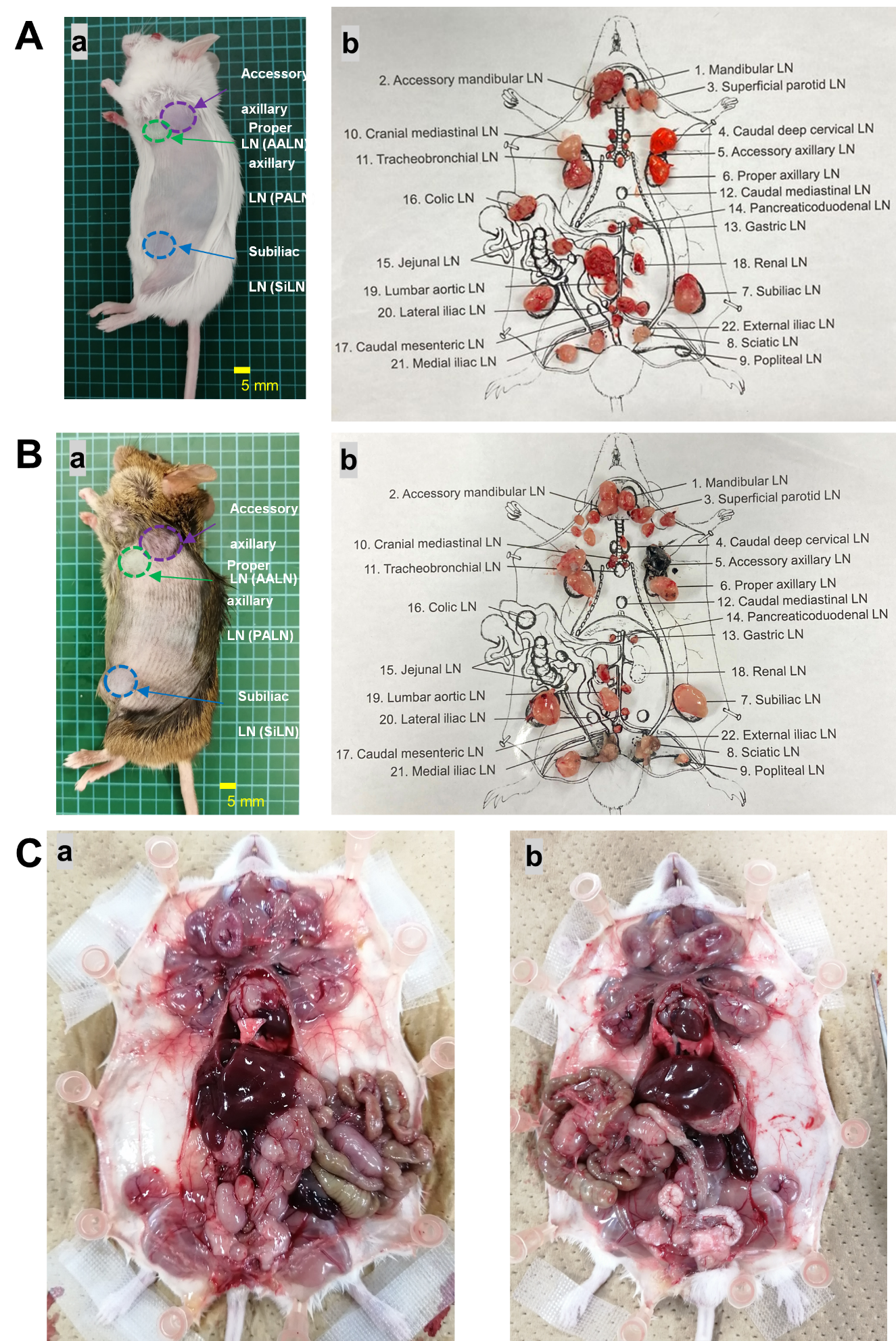


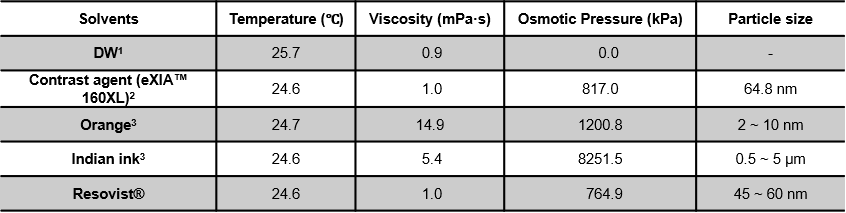


**D**

**Figure S1. Characteristics of the study animals and their LN localizations.** (A) Example of an MXH10/Mo/lpr mouse (a) and localization of lymph node (LN) topology (b). Modified and quoted from Shao *et al* with permission from the copyright holder [14]. (B) Example of an MXH51/Mo/lpr mouse (a) and localization of LN topology (b). Modified and quoted from Shao *et al* with permission from the copyright holder [14]. (C) Dissected male (a) and female (b) mouse used for illustration. (D) Parameters of tracers. ^1^Control for the measurement; ^2^copied from the manufacturer’s site; ^3^four individual measurements were carried out for each tracer.


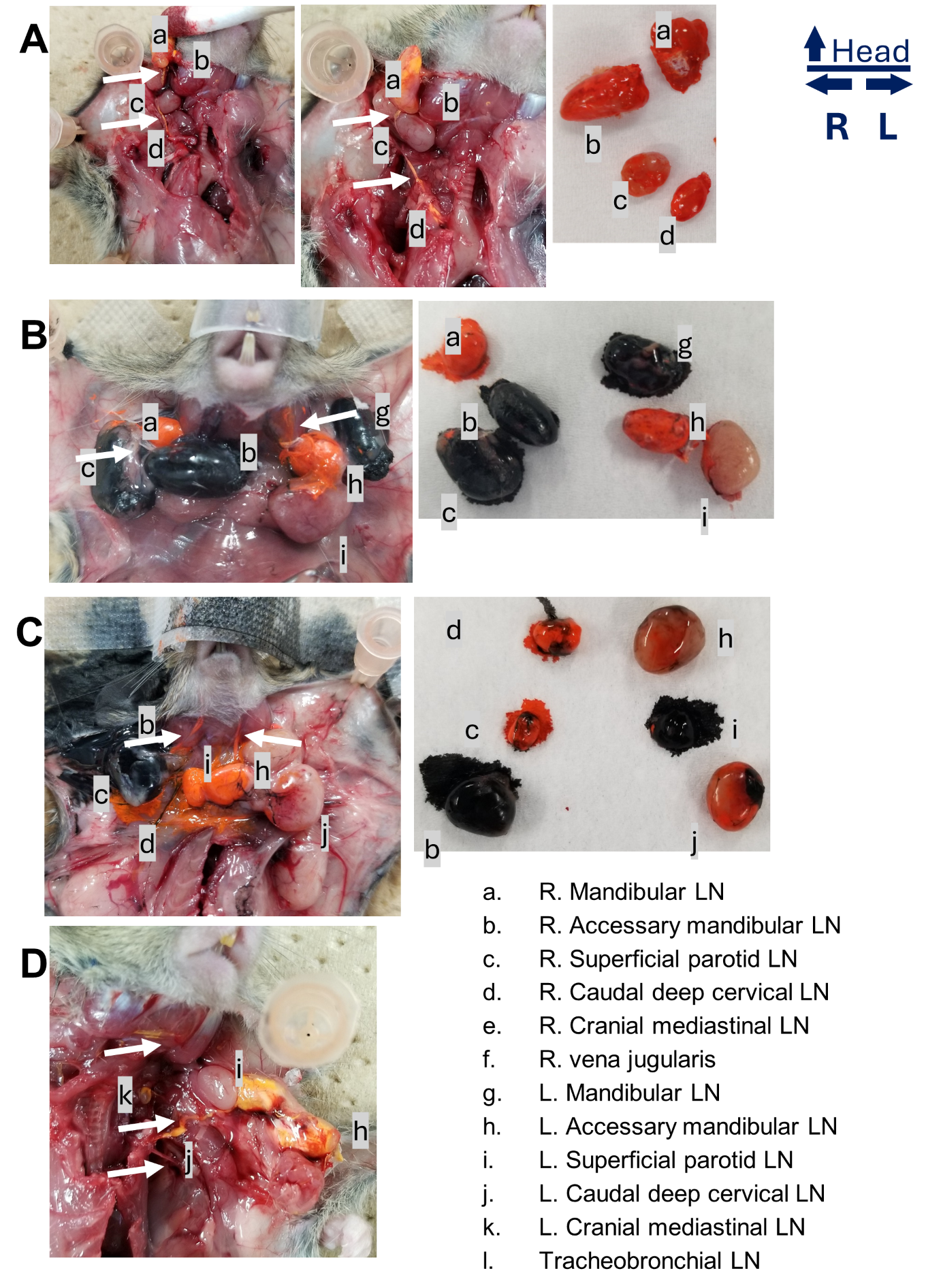


**
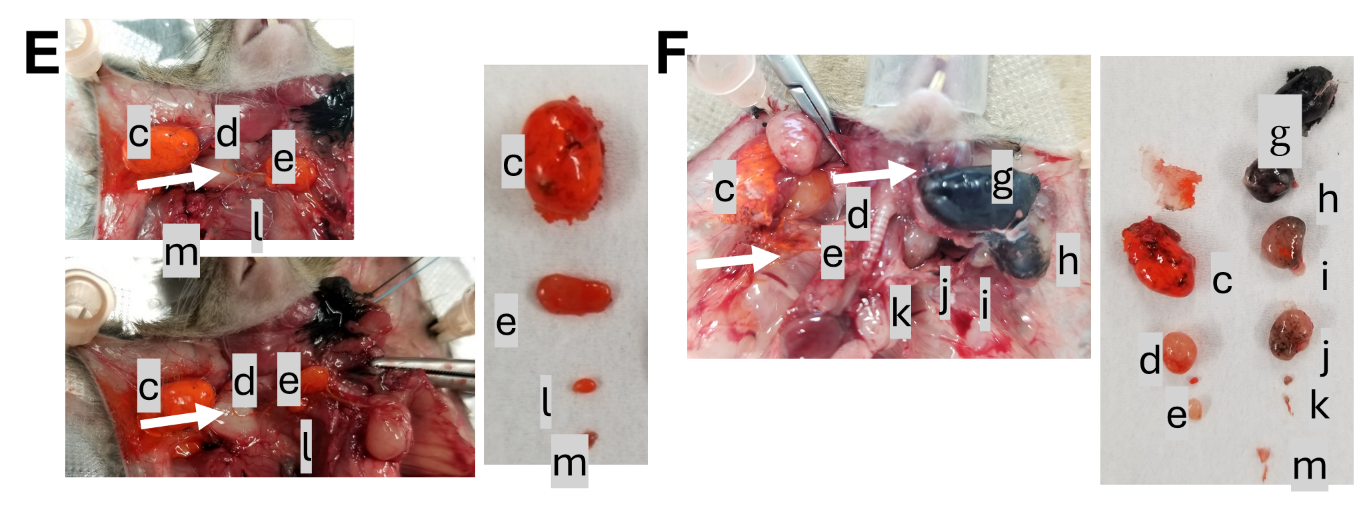
**

**Figure S2. The lymphatic flow patterns of head and neck region LNs.** Dye or ink was injected into the right (R.) or left (L.) indicated lymph nodes (LNs). a, R. mandibular lymph node (LN); b, R. accessary mandibular LN; c, R. superficial parotid LN; d, R. caudal deep cervical LN; e, R. cranial mediastinal LN; f, R. vena jugularis; g, L. mandibular LN; h, L. accessary mandibular LN; i, L. superficial parotid LN; j, L. caudal deep cervical LN; k, L. cranial mediastinal LN; l, tracheobronchial LN; m, caudal mediastinal LN; white arrow, lymphatic vessel. (A) Common flow pattern from the right or left mandibular LN. (B) Flow pattern of the right mandibular LN. (C) Common flow pattern from the right or left accessary mandibular LN. (D) The flow pattern of the left accessary mandibular LN. (E) Common flow pattern from the right or left accessary mandibular LN. (F) Flow pattern of the left accessary mandibular LN.


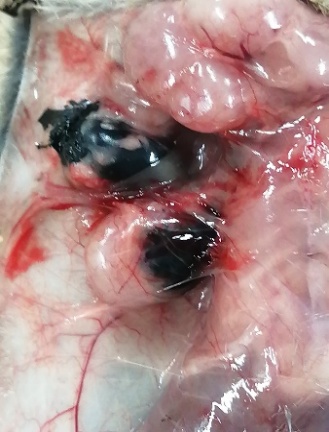


b

c


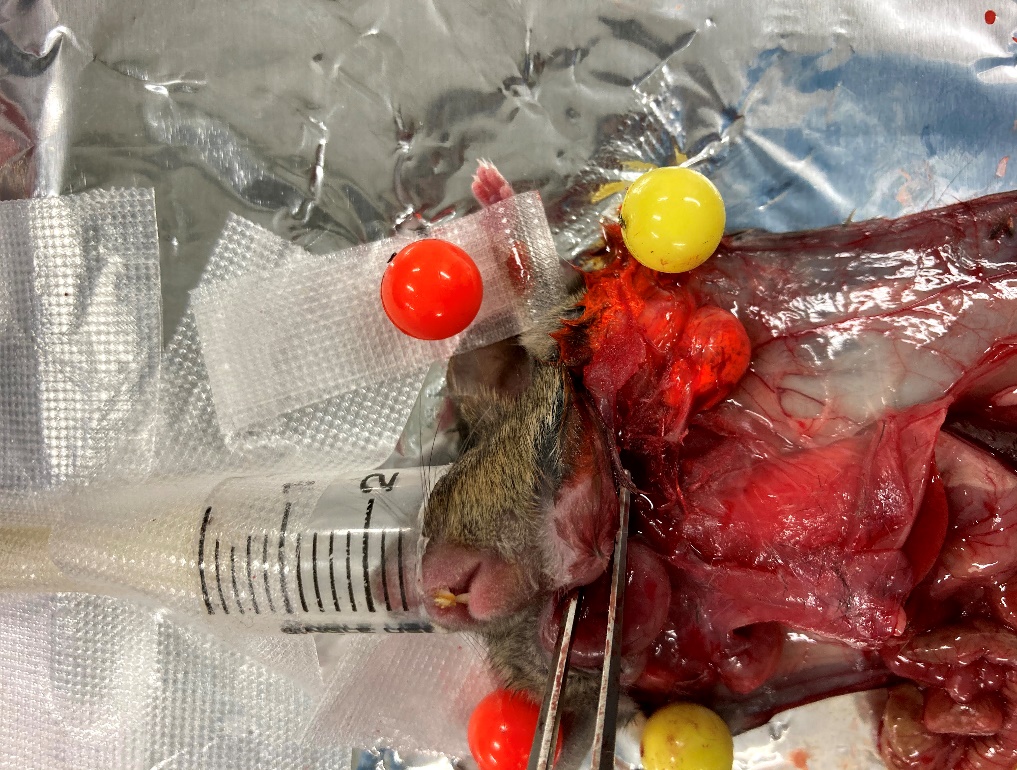


f

e


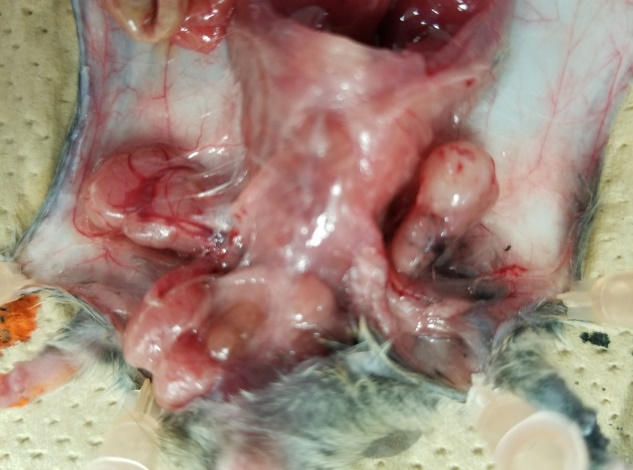


a

c

b


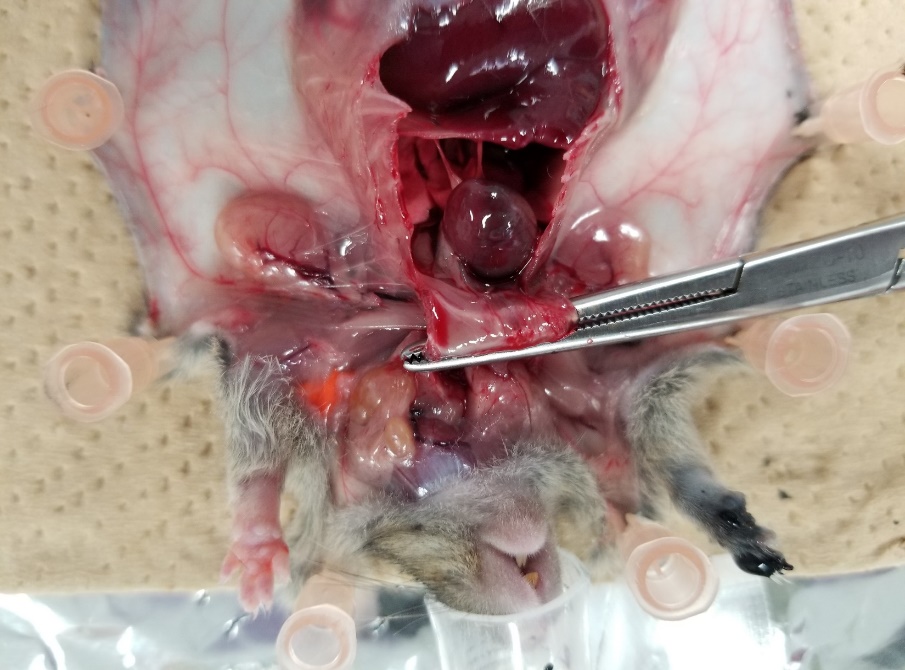


d

e

f


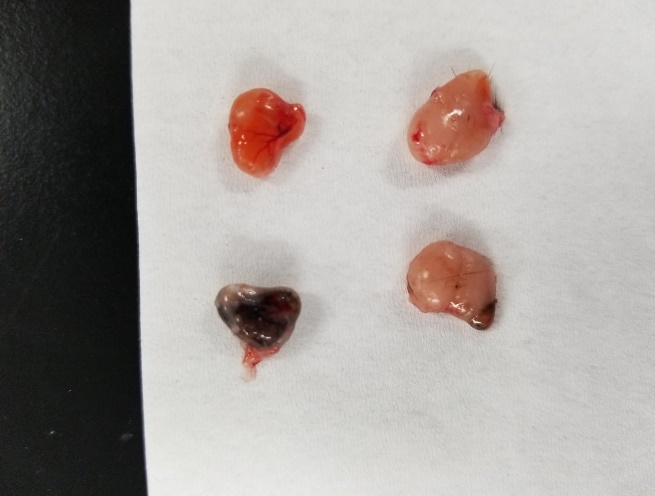

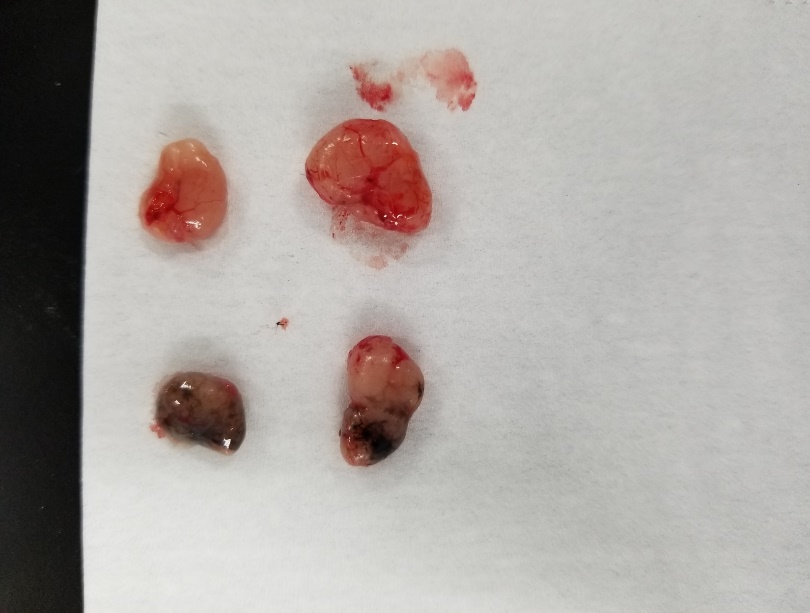


f

e

c

b

Head

**R**

**L**

**A**

**B**


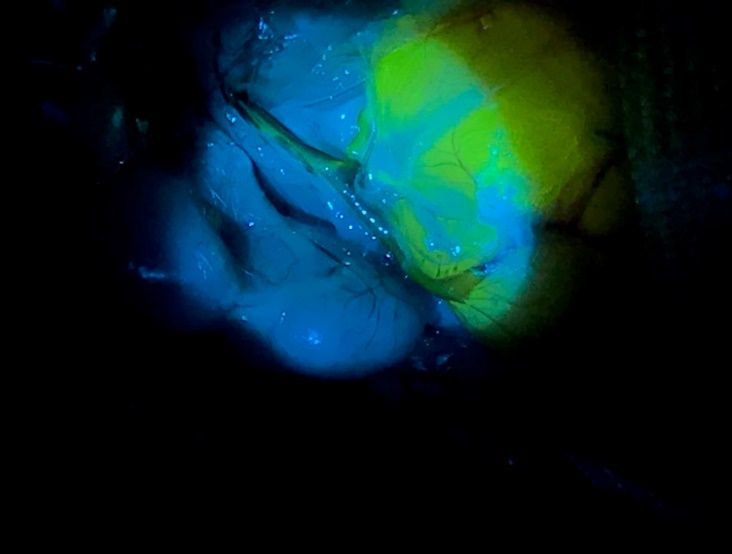


c

b

ELV (PALN)

Vein (AALN)

Vena subclavia


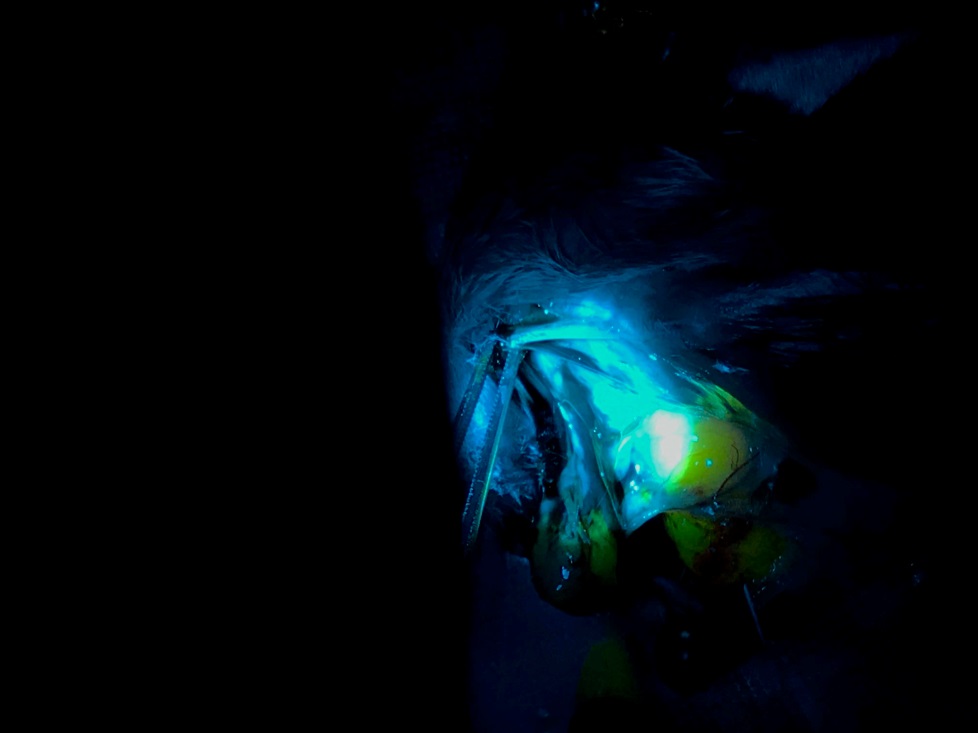


e

f

Vein (AALN)

ELV (PALN)

Vena subclavia

**C**

1. R. Forepaw
2. R. Accessary axillary LN
3. R. Proper axillary LN
4. L. Forepaw
5. L. Accessary axillary LN
6. L. Proper axillary LN

white arrow. Lymphatic vessel

ELV, efferent lymphatic vessel.

**Figure S3. The lymphatic flow patterns of upper limb region LNs.** Dye or ink was injected into the right or left indicated lymph nodes (LNs). a, right (R.) forepaw; b, R. accessary axillary LN; c, R. proper axillary LN; d, left (L.) forepaw; e, L. accessary axillary LN; f, L. proper axillary LN; white arrow, lymphatic vessel; ELV, efferent lymphatic vessel. (A) Flow pattern from the R. or L. forepaw. (B) Flow pattern from the R. or L. accessary axillary LN. (C) Investigation of the lymphatic vessel and lymph flow to the systemic circulation.


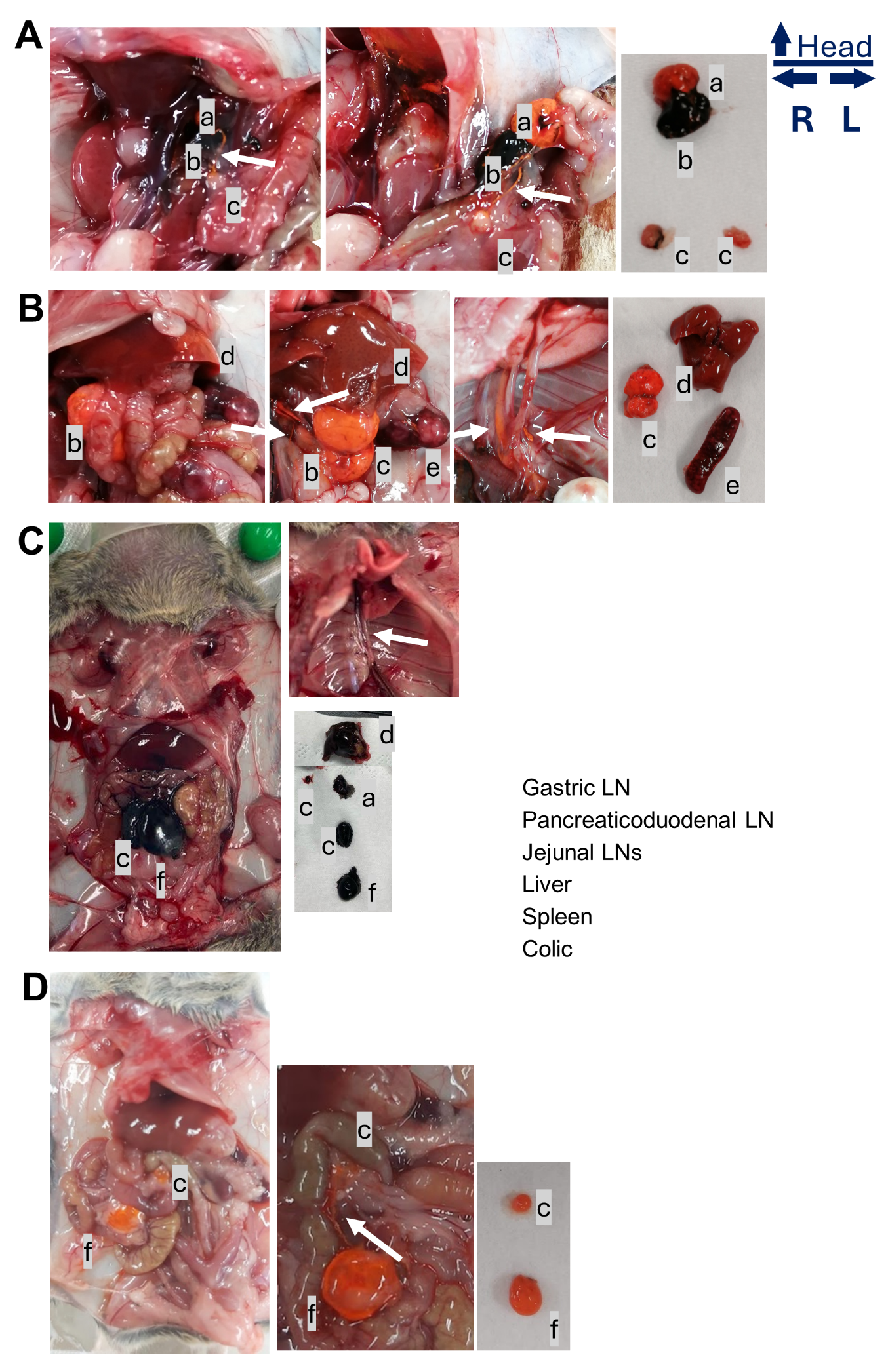


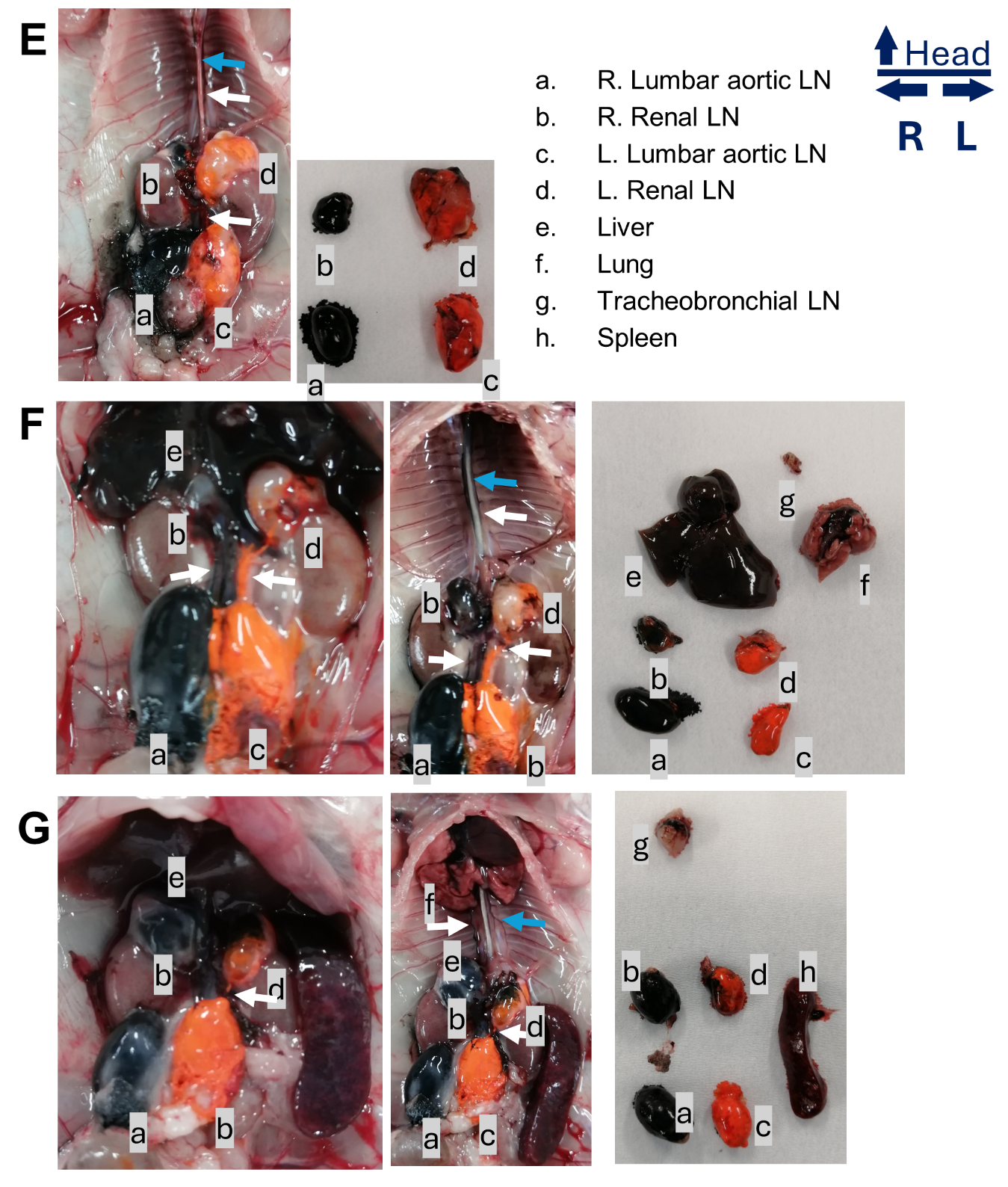


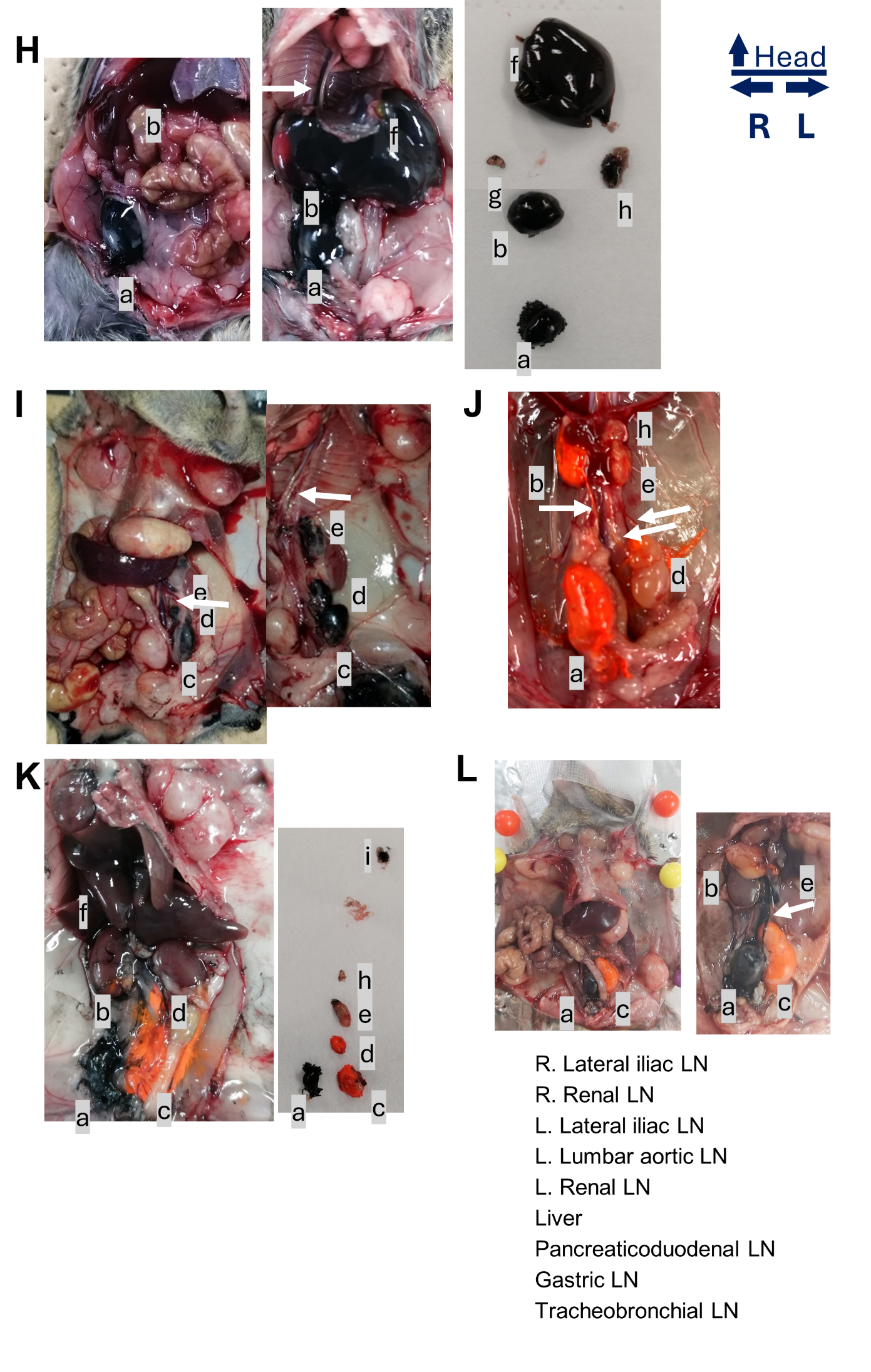


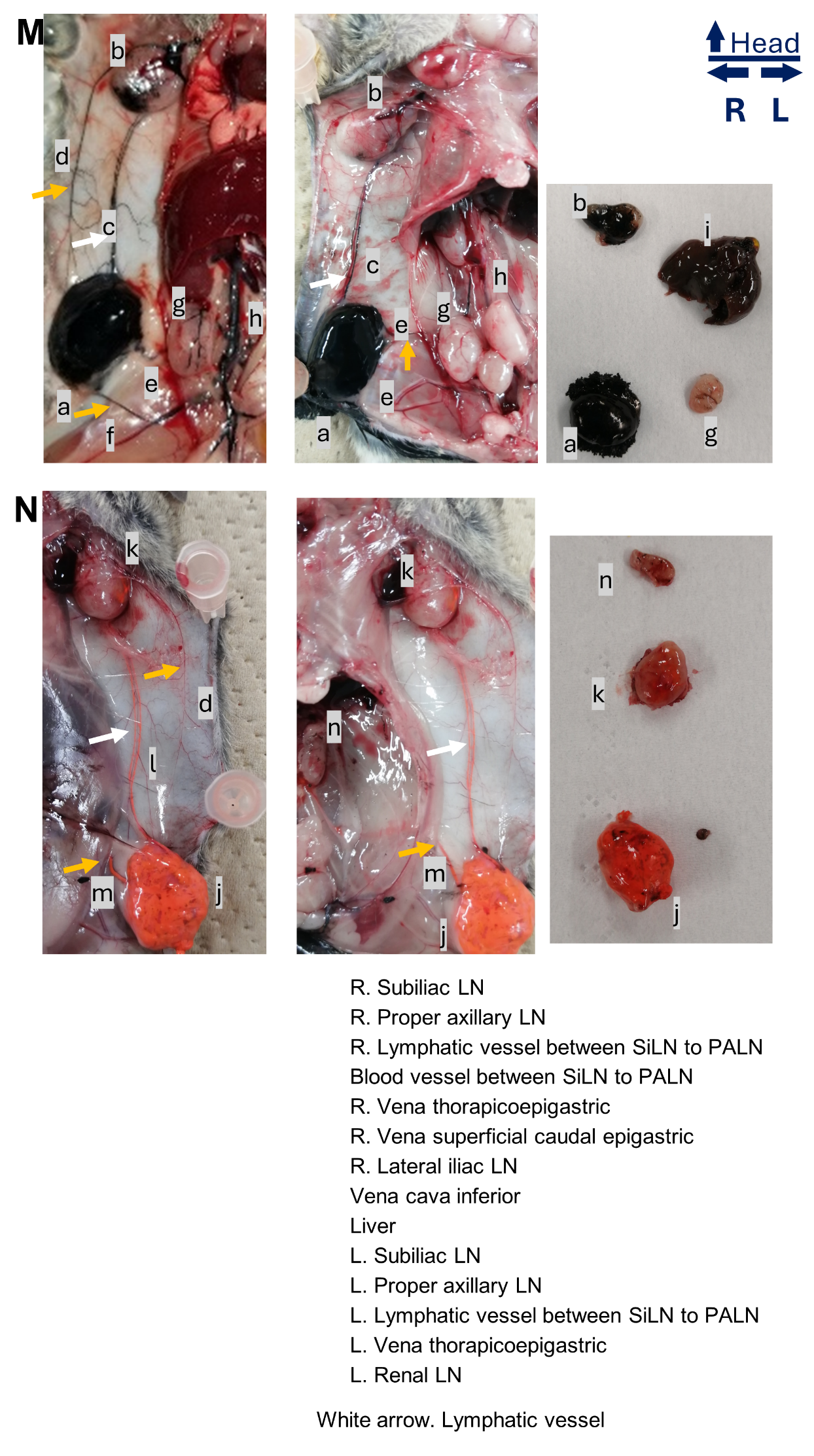


**Figure S4. The** **lymphatic flow patterns of abdominal region LNs.** Dye or ink was injected into the indicated lymph nodes (LNs). (A) Flow pattern from gastric LN (a, gastric LN; b, pancreaticoduodenal LN; c, jejunal LNs; d, liver; e, spleen; f, colic LN; white arrow, lymphatic vessel). (B) Flow pattern from pancreaticoduodenal LN (a, gastric LN; b, pancreaticoduodenal LN; c, jejunal LNs; d, liver; e, spleen; f, colic LN; white arrow, lymphatic vessel). (C, D) Flow pattern from colic LN (a, gastric LN; b, pancreaticoduodenal LN; c, jejunal LNs; d, liver; e, spleen; f, colic LN; white arrow, lymphatic vessel). (E–G) Flow pattern from the right (R.) or left (L.) lumbar aortic LN (a, R. lumbar aortic LN; b, R. renal LN; c, L. lumbar aortic LN; d, L. renal LN; e, liver; f, lung; g, tracheobronchial LN; h, spleen; white arrow, lymphatic vessel; blue arrow, blood vessel). (H–L) Flow pattern from the right (R.) or left (L.) lateral iliac LN (a, R. lateral iliac LN; b, R. renal LN; c, L. lateral iliac LN; d, L. lumbar aortic LN; e, L. renal LN; f, liver; g, pancreaticoduodenal LN; h, gastric LN; i, tracheobronchial LN; white arrow, lymphatic vessel). (M, N) Flow pattern from the right or left subiliac LN (siLN) (a, R. subiliac LN; b, R. proper axillary LN; c, R. lymphatic vessel between the SiLN routed to the para-aortic lymph node (PALN); d, blood vessel running between the SiLN and PALN; e, R. vena thoracoepigastric; f, vena superficial caudal epigastric; g, R. lateral iliac LN; h, vena cava inferior; i, liver; j, L. subiliac LN; k, L. proper axillary LN; l, L. lymphatic vessel between SiLN to PALN; m, L. vena thoracoepigastric; n, L. renal LN; white arrows, lymphatic vessels; orange arrows, blood vessels).


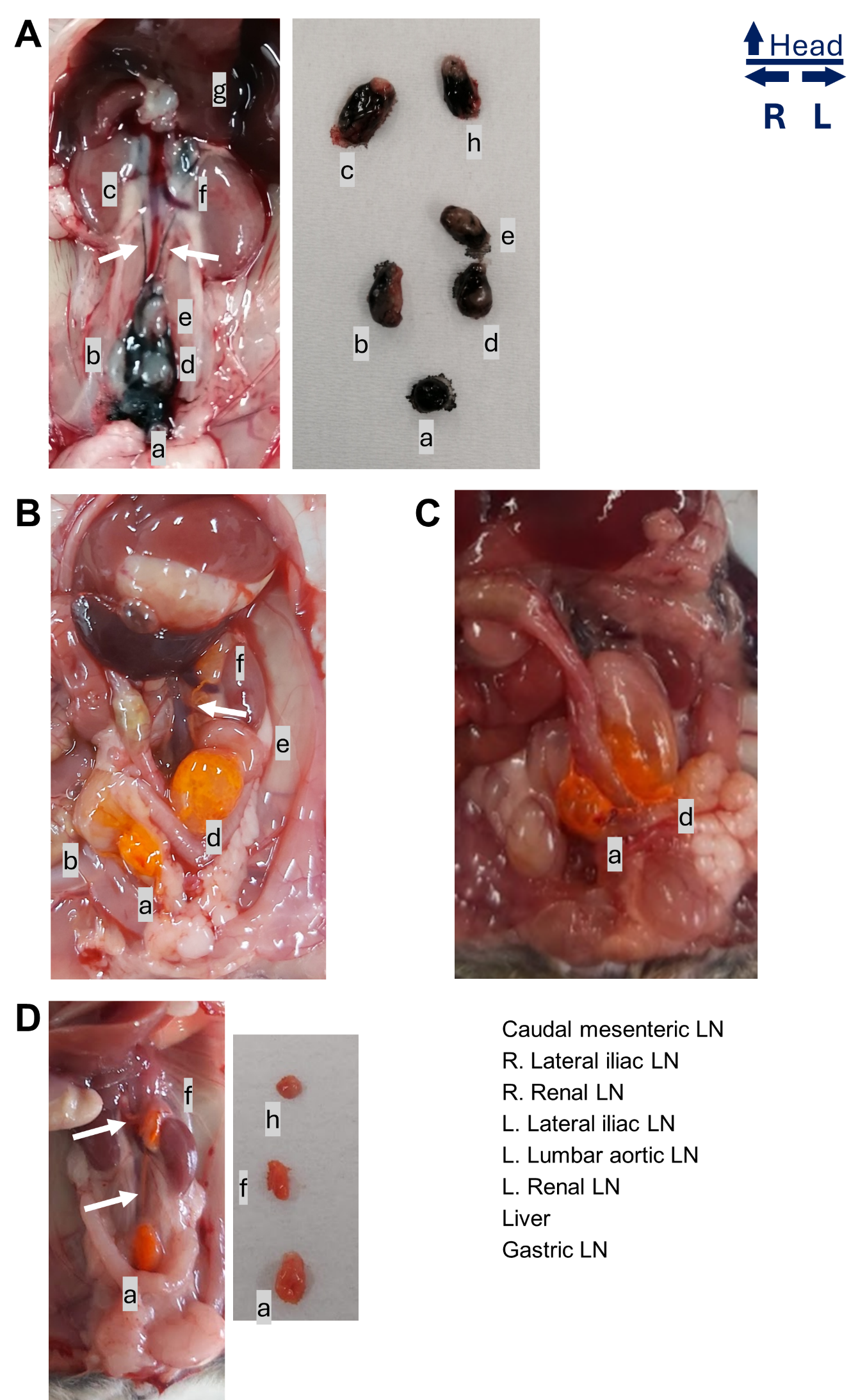


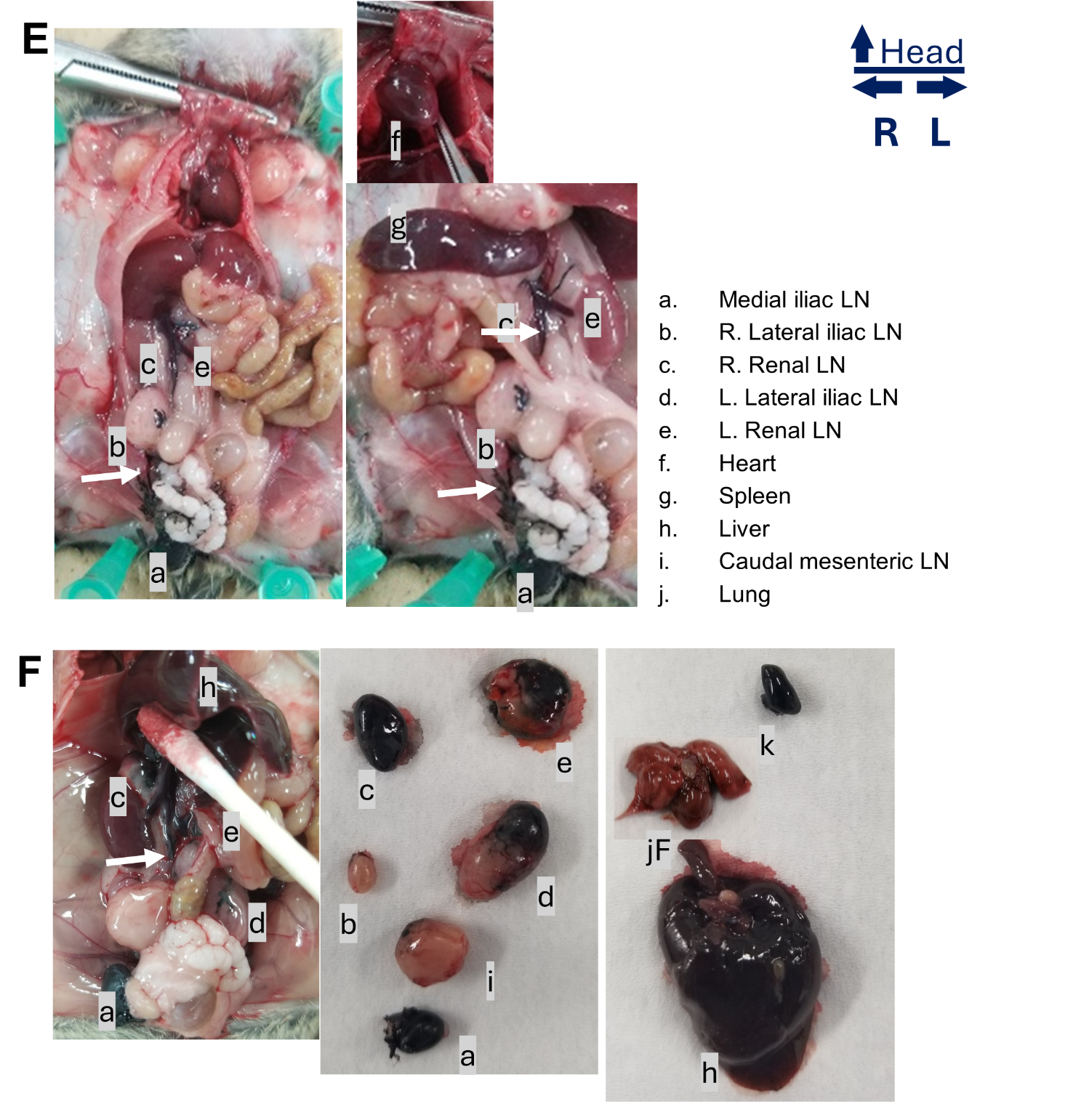


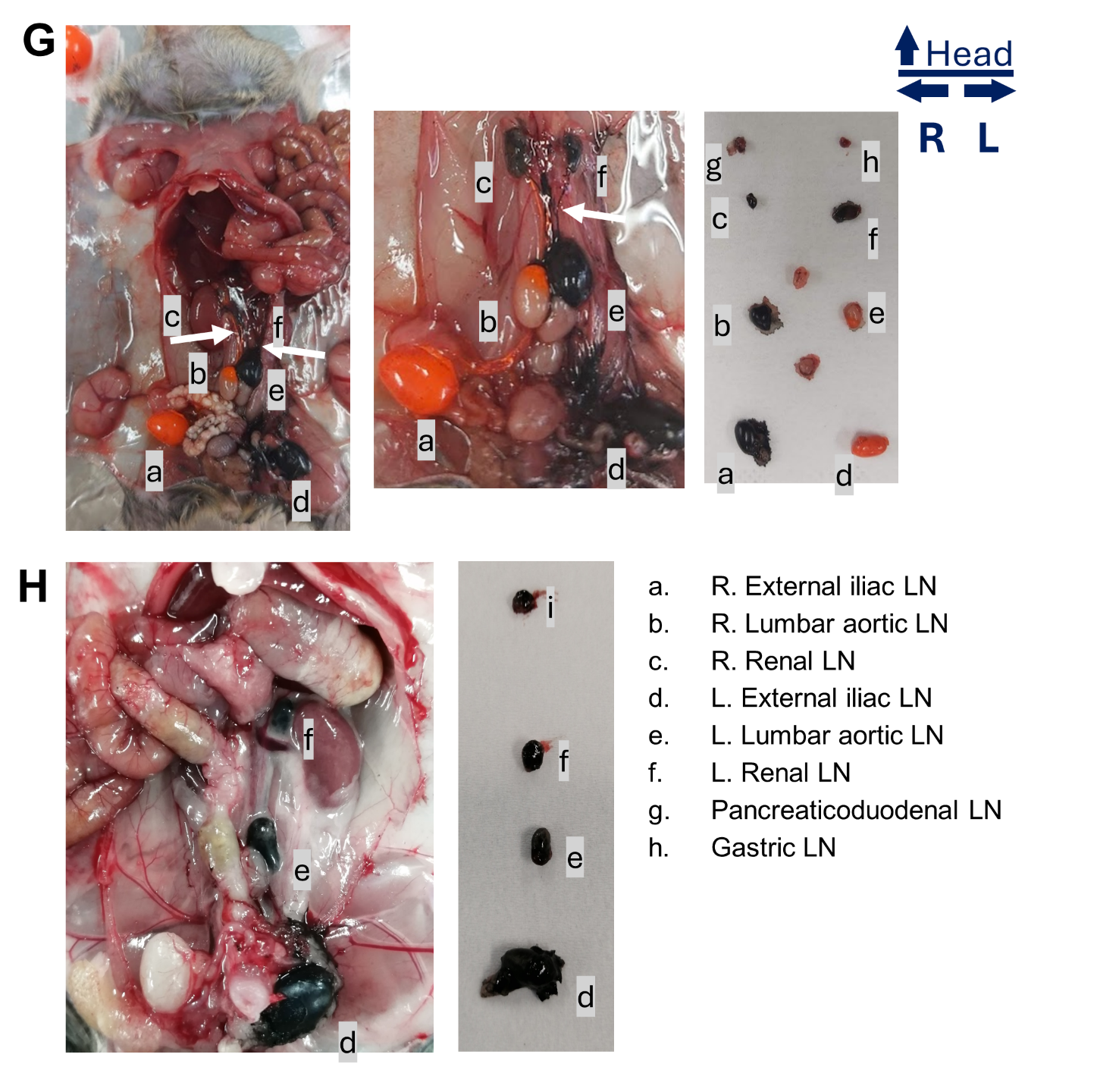


**
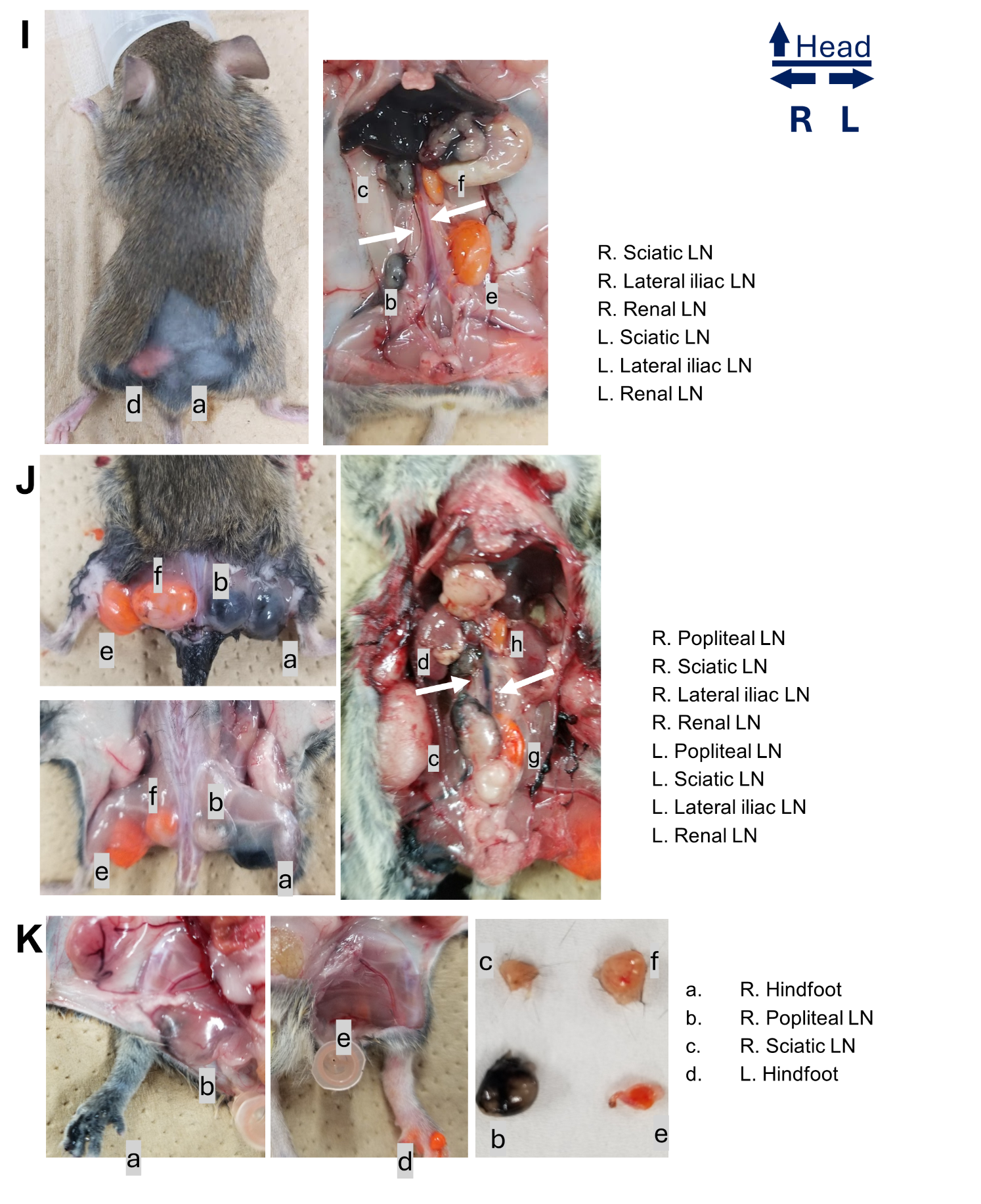
**

**Figure S5. The lymphatic flow patterns of lower limb region LNs.** Dye or ink was injected into the indicated lymph nodes (LNs). (A–D) Flow pattern from caudal mesenteric LN (a, caudal mesenteric LN; b, right (R.) lateral iliac LN; c, R. renal LN; d, left (L.) lateral iliac LN; e, L. lumbar aortic LN; f, L. renal LN; g, liver; h, gastric LN; white arrow, lymphatic vessel). (E, F) Flow pattern from medial iliac LN (a, medial iliac LN; b, R. lateral iliac LN; c, R. renal LN; d, L. lateral iliac LN; e, L. renal LN; f, heart; g, spleen; h, liver; i, caudal mesenteric LN; j, lung; k, caudal mediastinal LN; white arrow, lymphatic vessel). (G, H) Flow pattern from the right or left external iliac LN (a, R. external iliac LN; b, R. lumbar aortic LN; c, R. renal LN; d, L. external iliac LN; e, L. lumbar aortic LN; f, L. renal LN; g, pancreaticoduodenal LN; h, gastric LN; i, tracheobronchial LN; white arrow, lymphatic vessel). (I) Flow pattern from the right or left sciatic LN (a, R. sciatic LN; b, R. lateral iliac LN, c, R. renal LN; d, L. sciatic LN; e, L. lateral iliac LN; f, L. renal LN; white arrow, lymphatic vessel). (J) Flow pattern from the right or left popliteal LN (a, R. popliteal LN; b, R. sciatic LN; c, R. lateral iliac LN; d, R. renal LN; e, L. popliteal LN; f, L. sciatic LN; g, L. lateral iliac LN; h, L. renal LN; white arrow, lymphatic vessel). (K) Flow pattern from the right or left hindfoot (a, R. hindfoot; b, R. popliteal LN; c, R. sciatic LN; d, L. hindfoot; e, L. popliteal LN; f, L. sciatic LN).

**
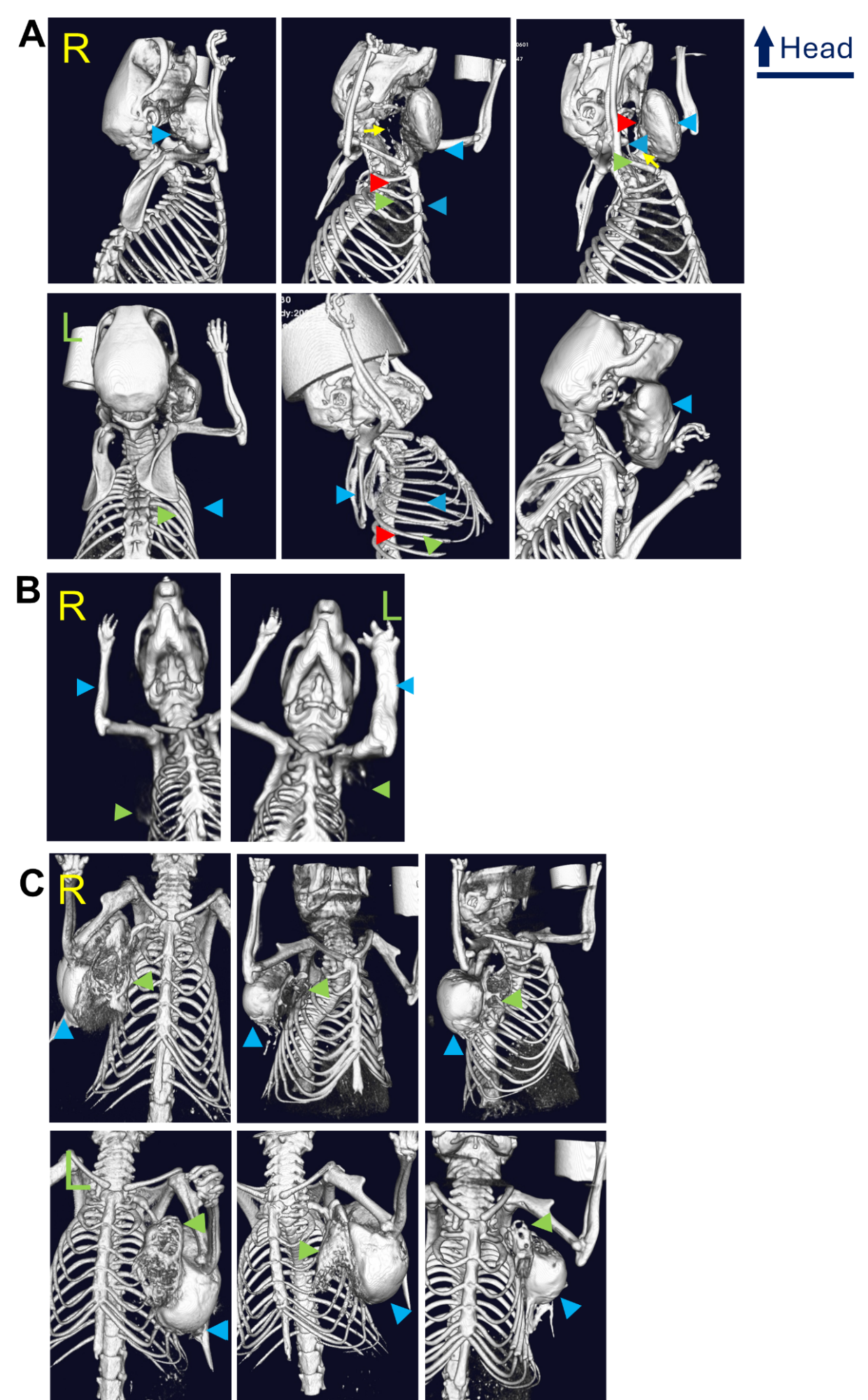
**

**Figure S6.** **Lymphatic tracing of head and neck region and upper limb region LNs (CT).** (A) Contrast agent injected into right or left mandibular LN. The flow of the contrast agent was recorded at 5 min after injection within 5-min intervals. R, right; L, left; blue arrowhead, injection site (mandibular LN); red arrowhead, lymphatic vessel between mandibular LN and accessory mandibular LN; yellow arrowhead, accessory mandibular LN; green arrowhead, lymphatic vessel between accessory mandibular LN and superficial parotid LN; yellow arrow, lymphatic vessel runs next to cervical vertebrae. (B) Contrast agent injected into the right or left forepaw. The flow of the contrast agent was recorded 5 min after injection at 5-min intervals. R, right; L, left; blue arrowhead, injection site (forepaw); green arrowhead, accessory axillary LN. (C) Contrast agent injected into the right or left accessary axillary LN. The flow of the contrast agent was recorded 5 min after injection at 5-min intervals. R, right; L, left; blue arrowhead, injection site (accessory axillary LN); green arrowhead, proper axillary LN.


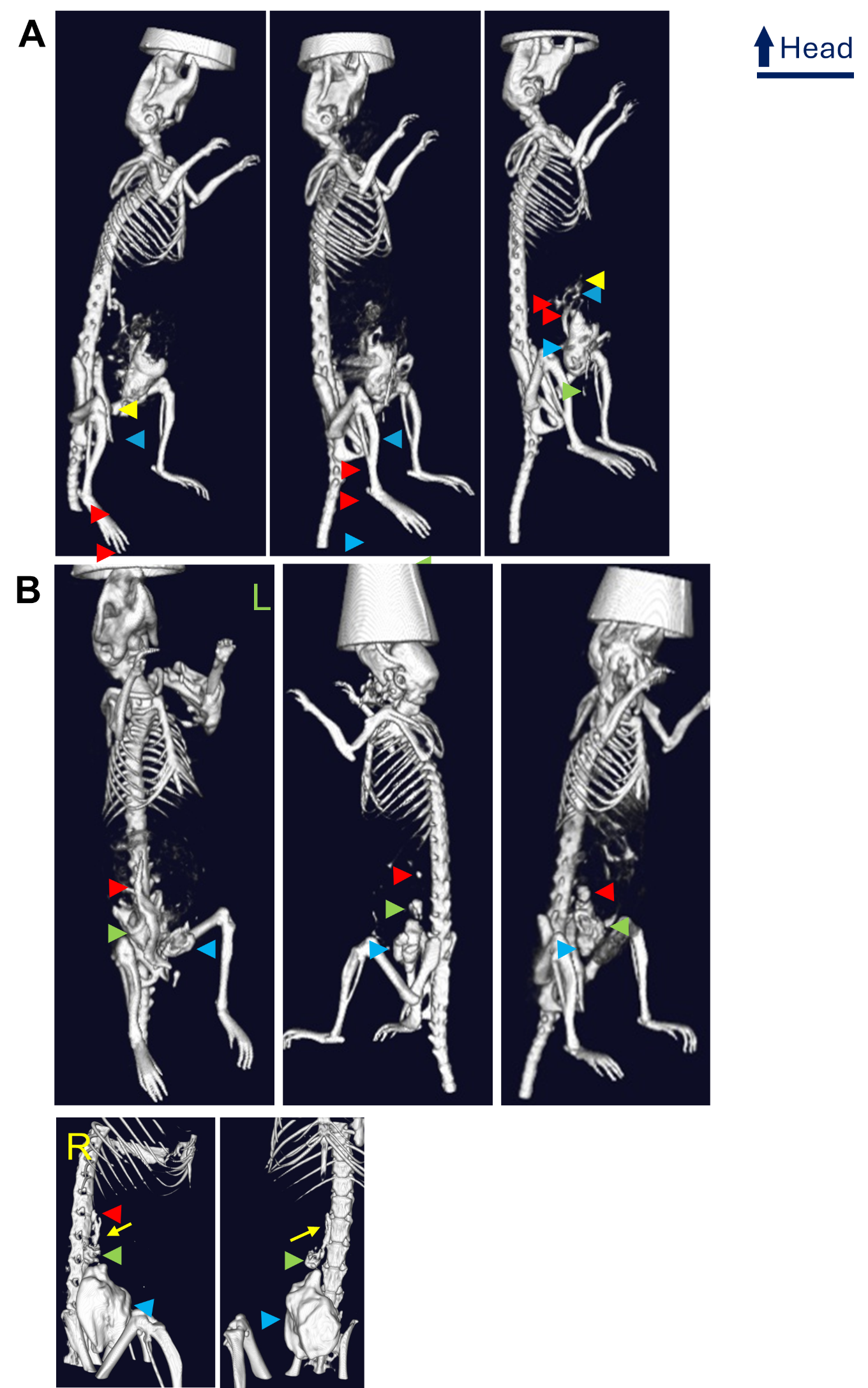


**
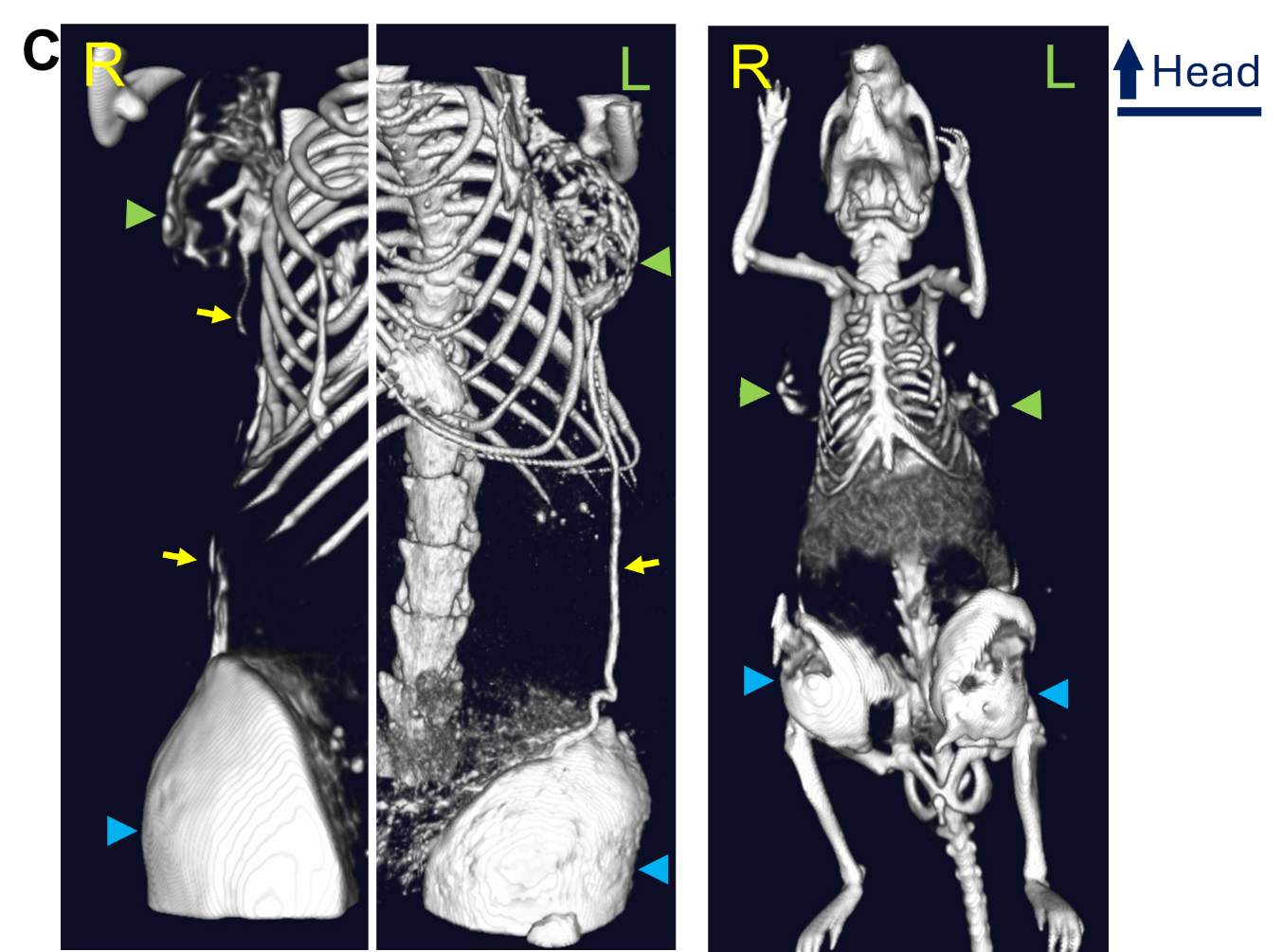
**

**Figure S7. Lymphatic tracing of abdominal region LNs (CT).** (A) Contrast agent was injected into the colic lymph node (LN). The flow of the contrast agent was recorded 5 min after injection at 5-min intervals. Blue arrowhead, injection site (colic LN); red arrowhead, jejunal LNs; yellow arrowhead, renal LN; yellow arrowhead, gastric LN; green arrowhead, a trace of contrast agent. (B) Contrast agent was injected into the right or left lateral iliac LN. The flow of the contrast agent was recorded 5 min after injection at 5-min intervals. R, right; L, left; blue arrowhead, injection site (lateral iliac LN); green arrowhead, lumbar aortic LN; red arrowhead, renal LN; yellow arrow, lymphatic vessels upwards to the cardiorespiratory system. (C) Contrast agent injected into the right or left subiliac LN. The flow of the contrast agent was recorded 5 min after injection at 5-min intervals. Blue arrowhead, injection site (subiliac LN); green arrowhead, proper axillary LNs; yellow arrow, lymphatic vessel.


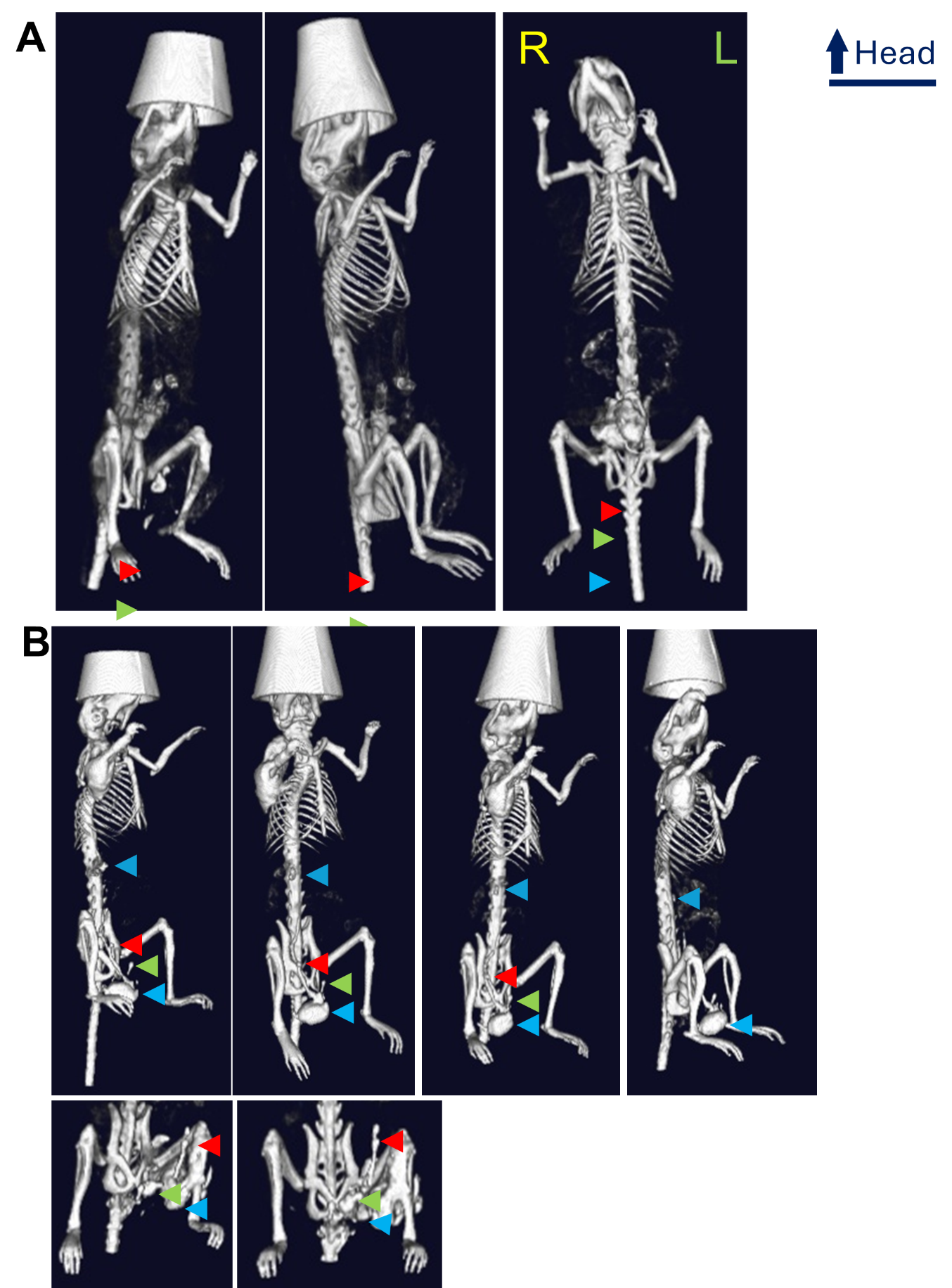


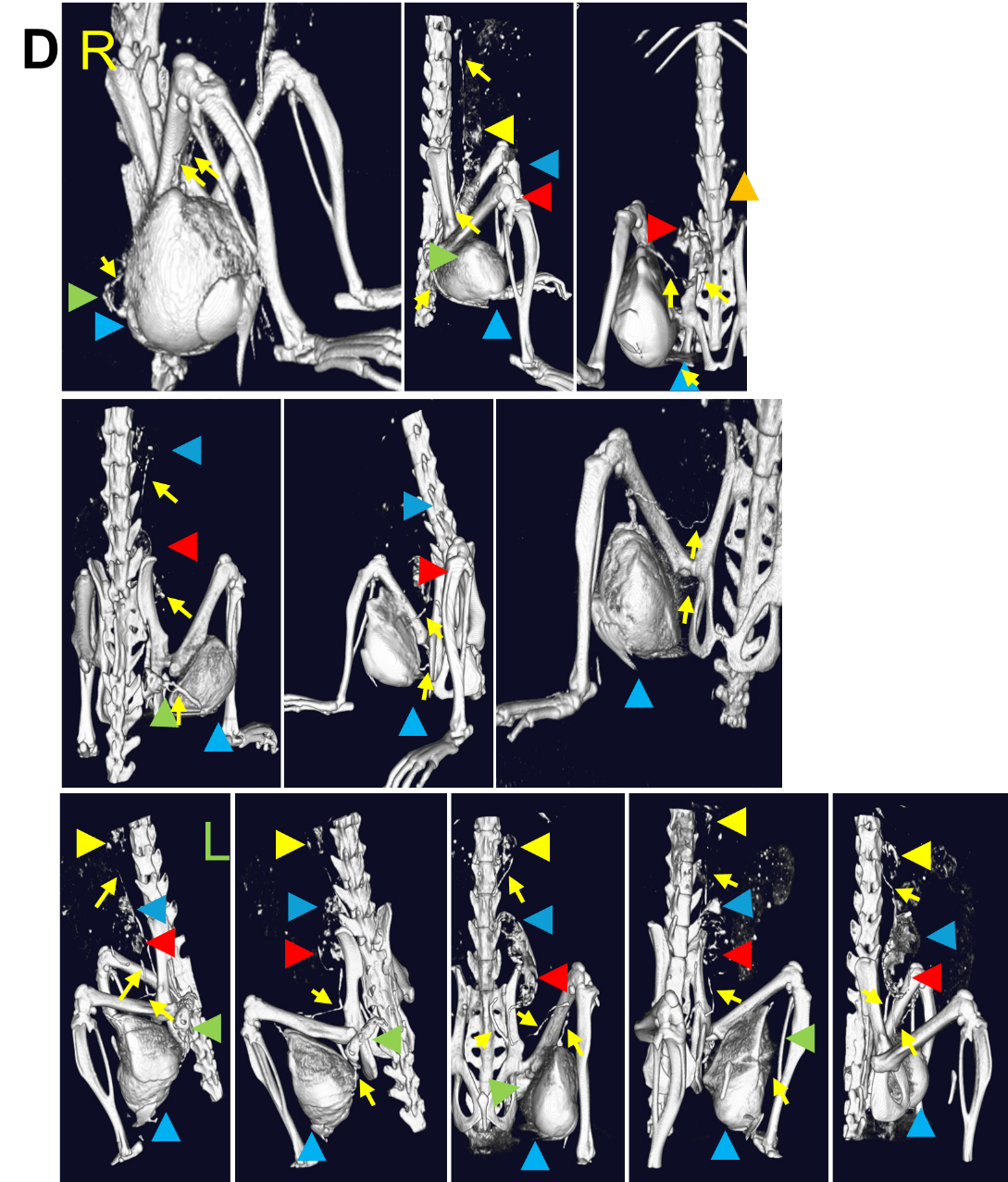


Head

**C**


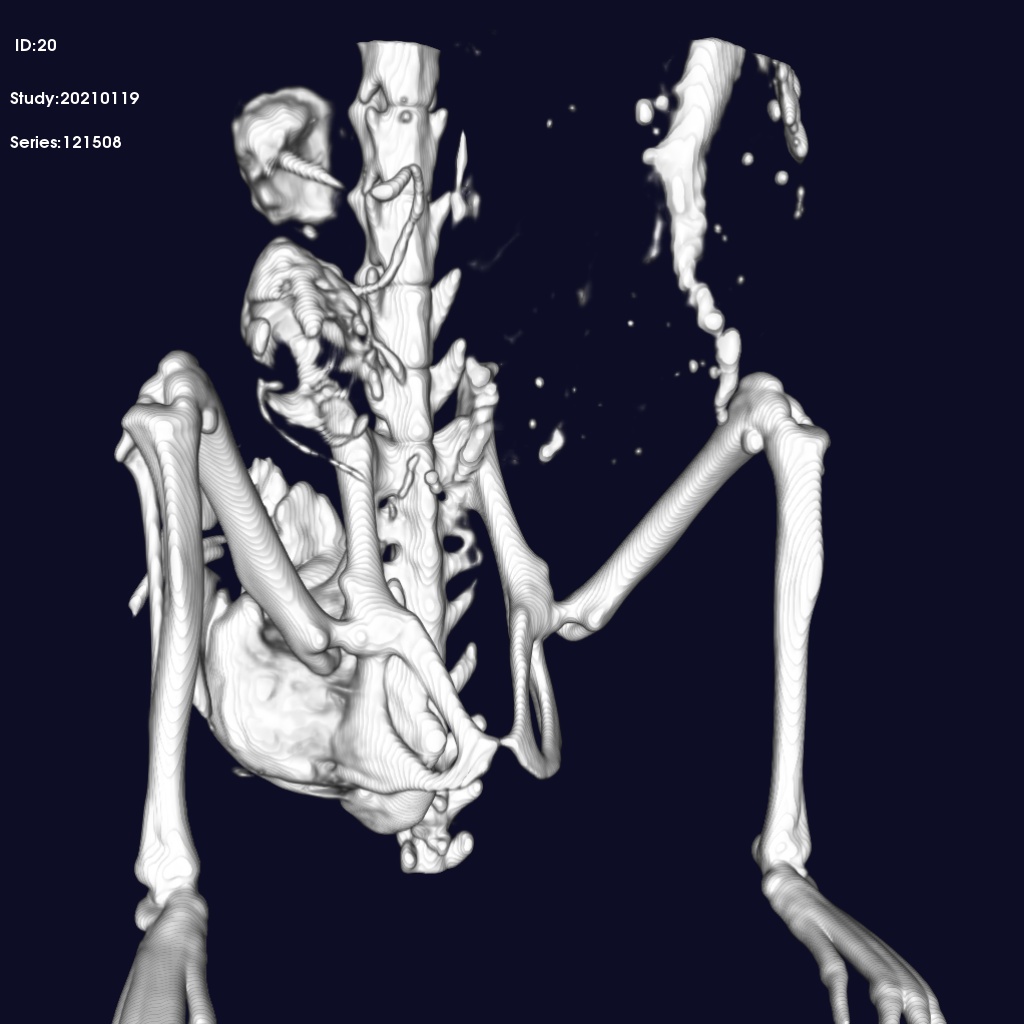

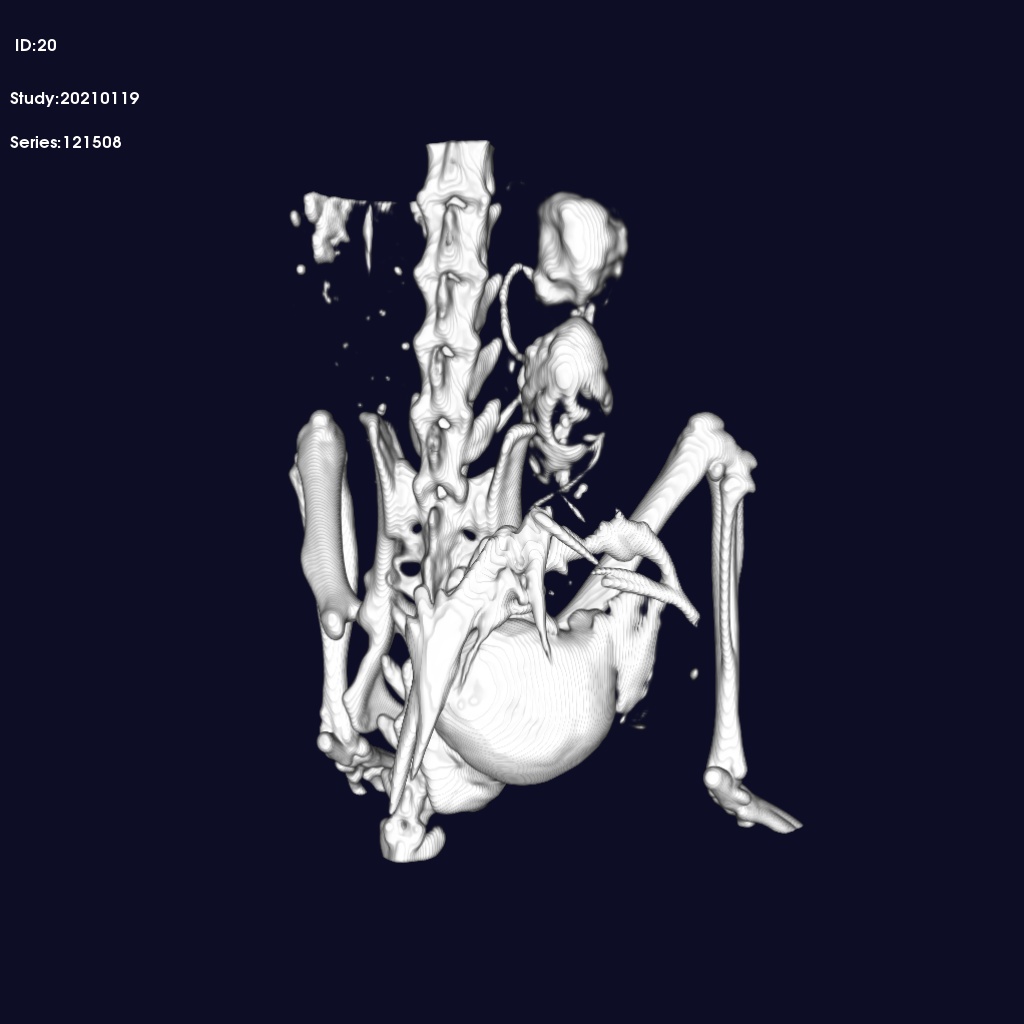

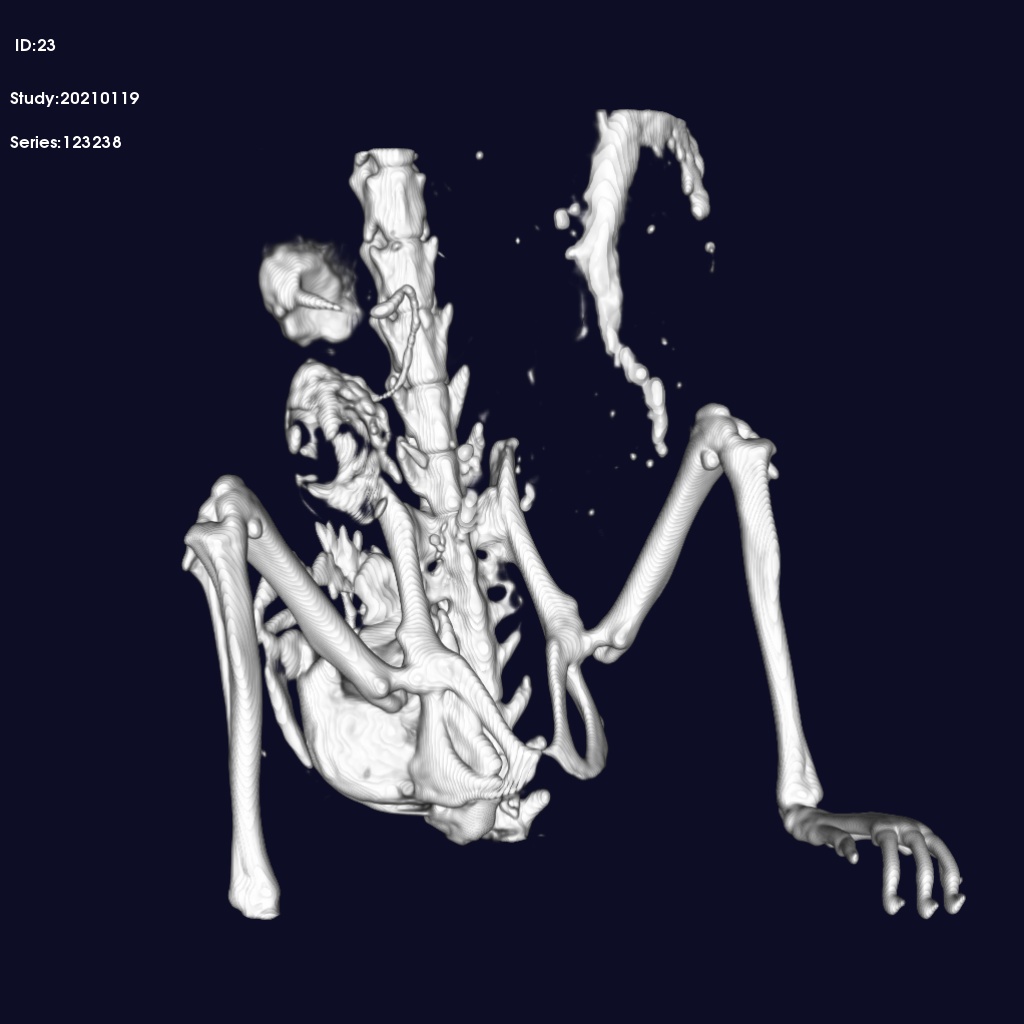

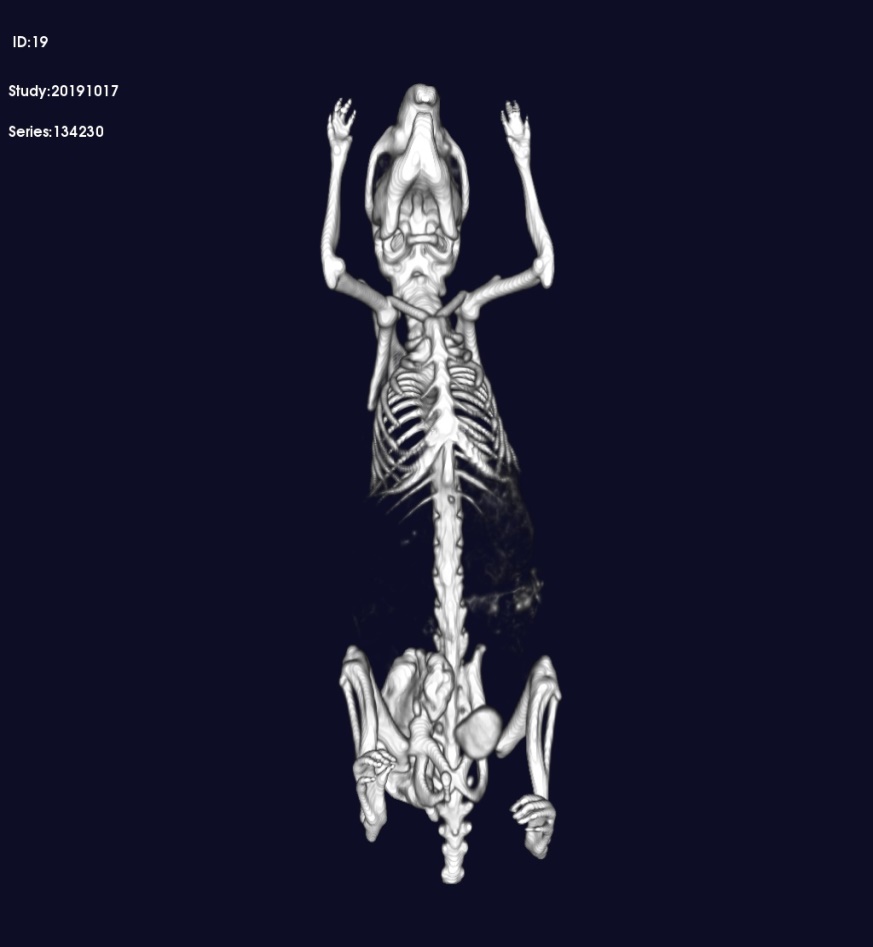


R


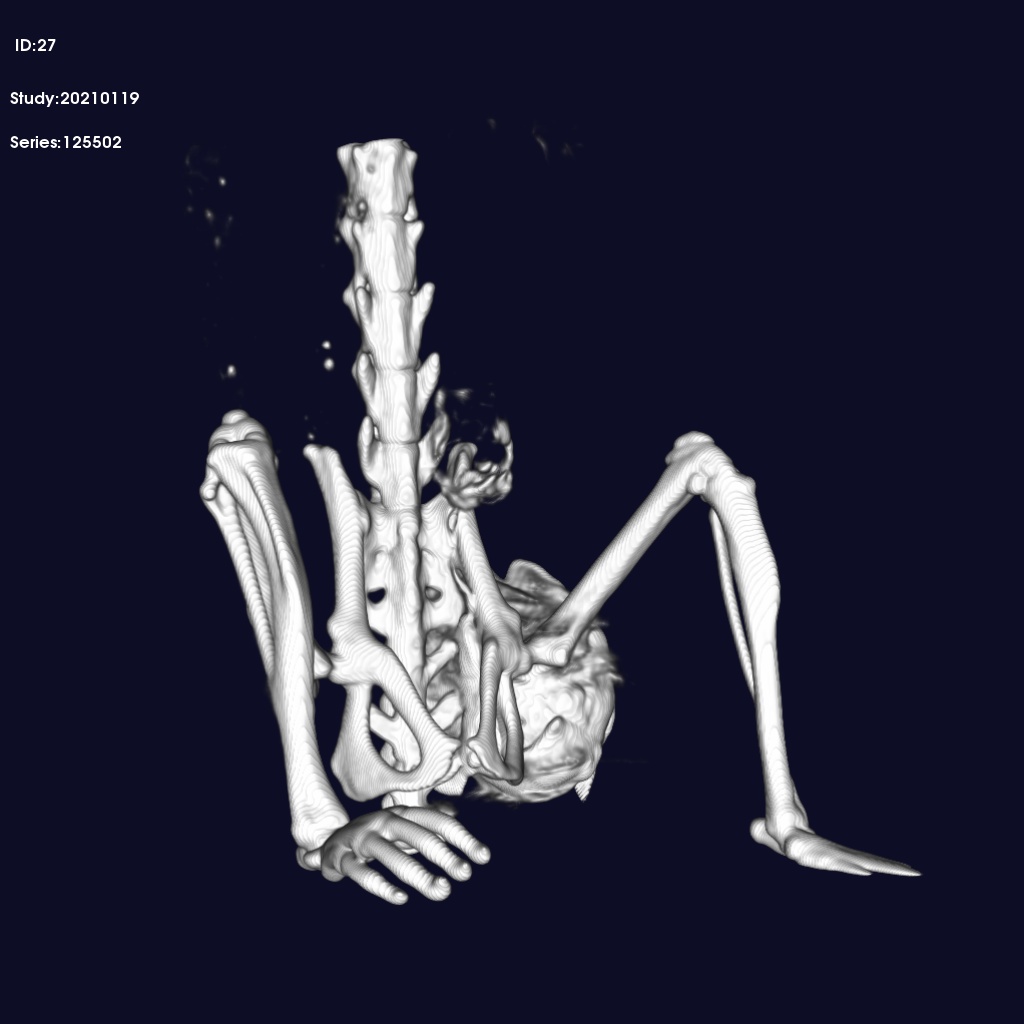

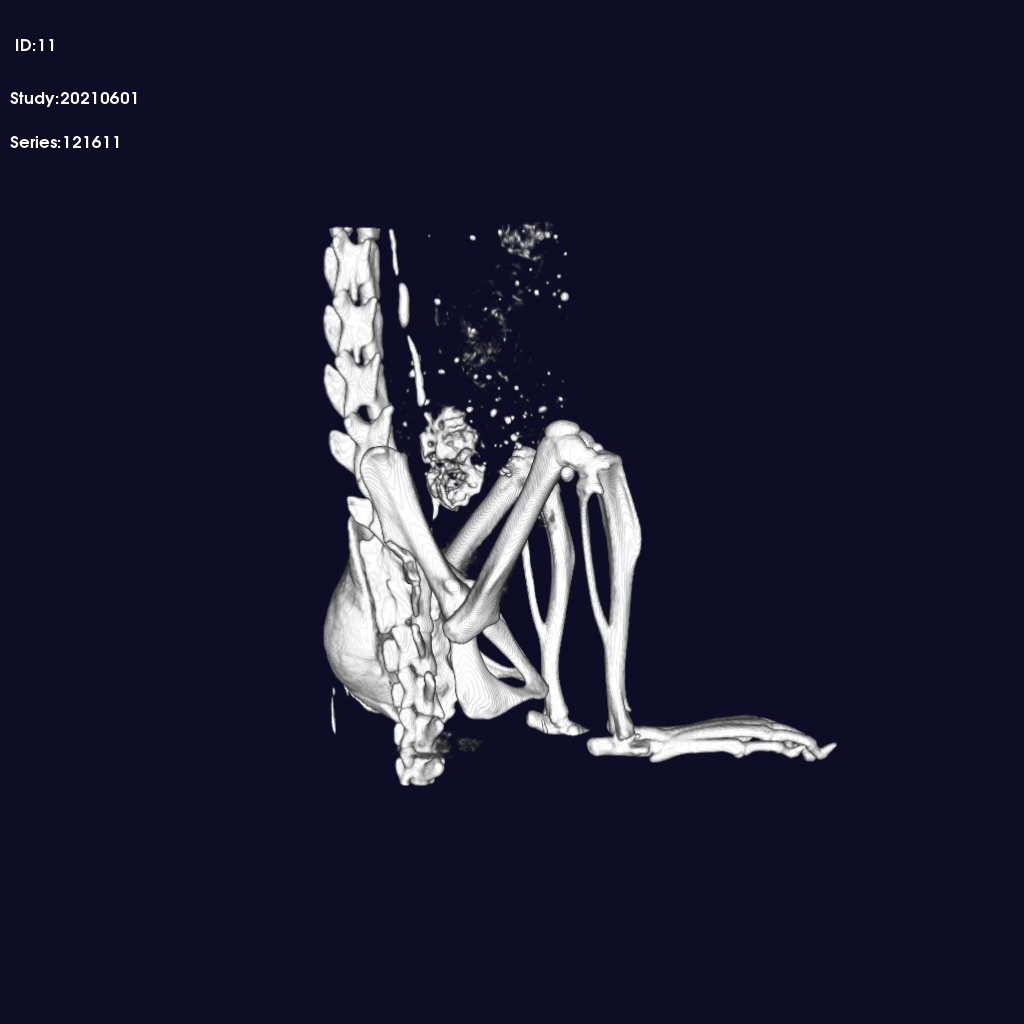

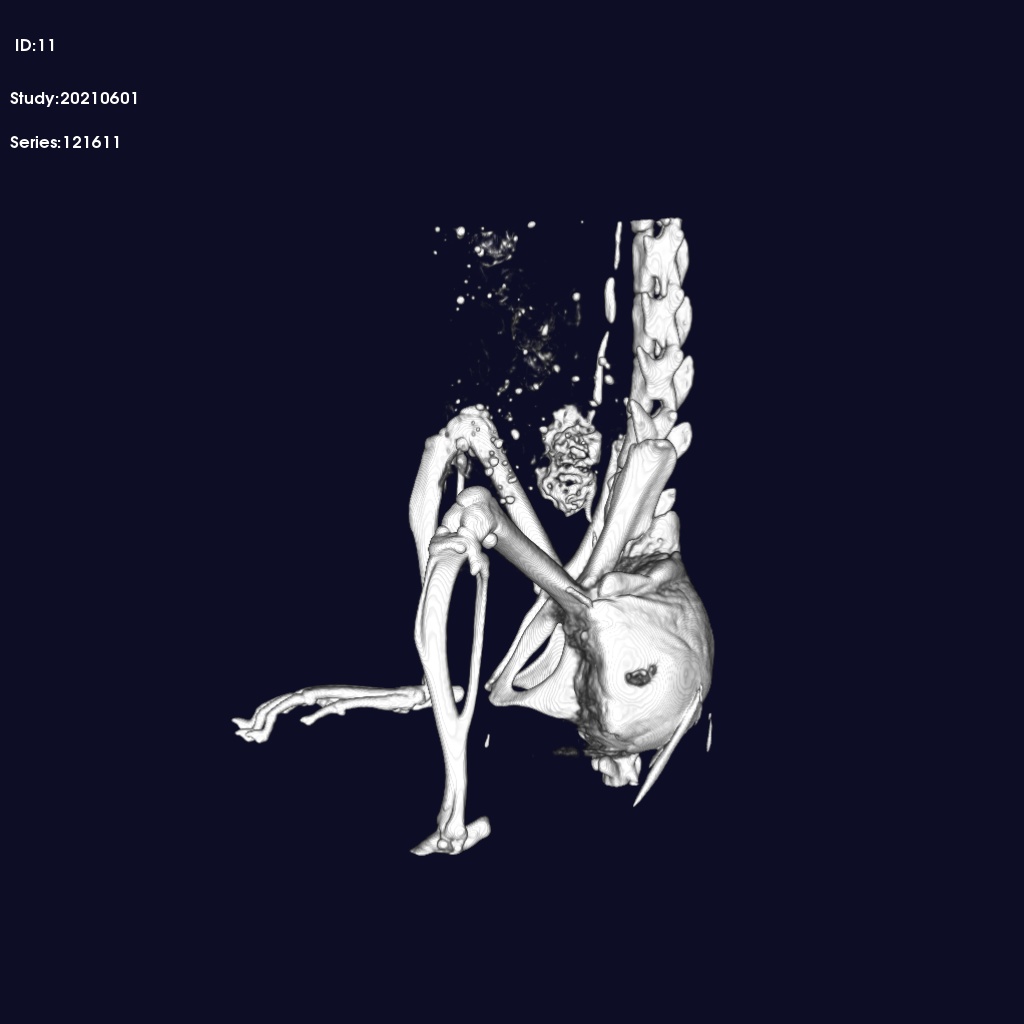


L


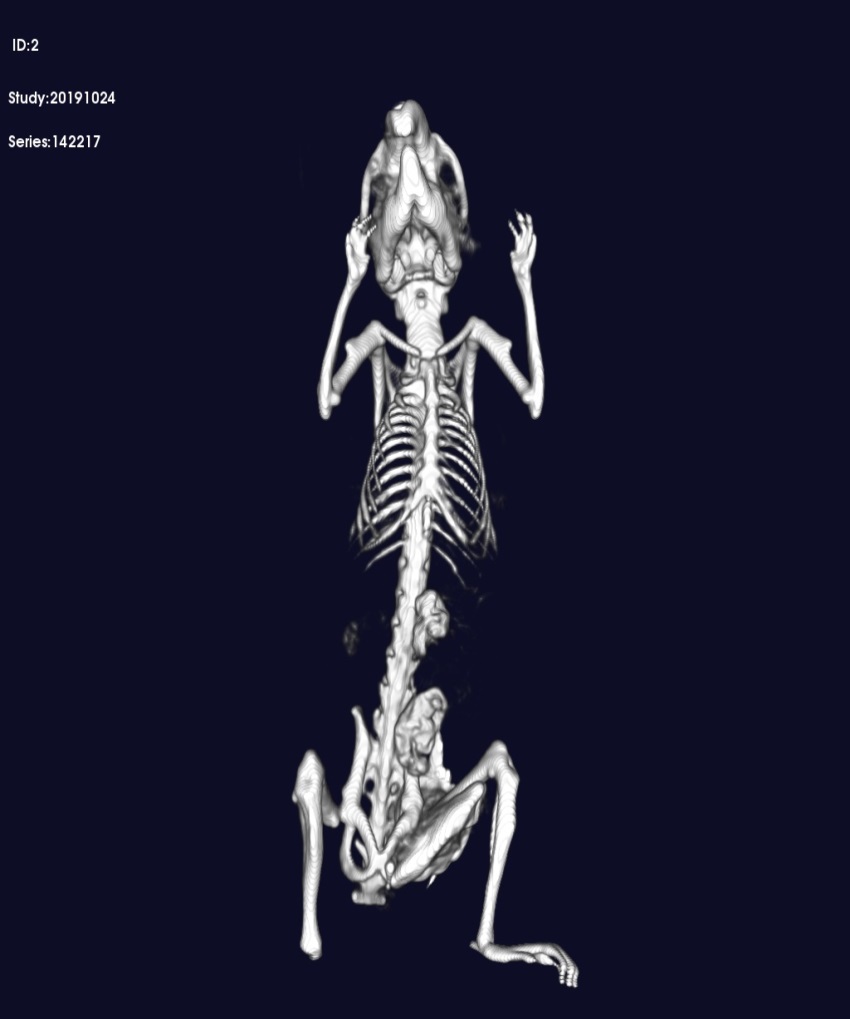


**
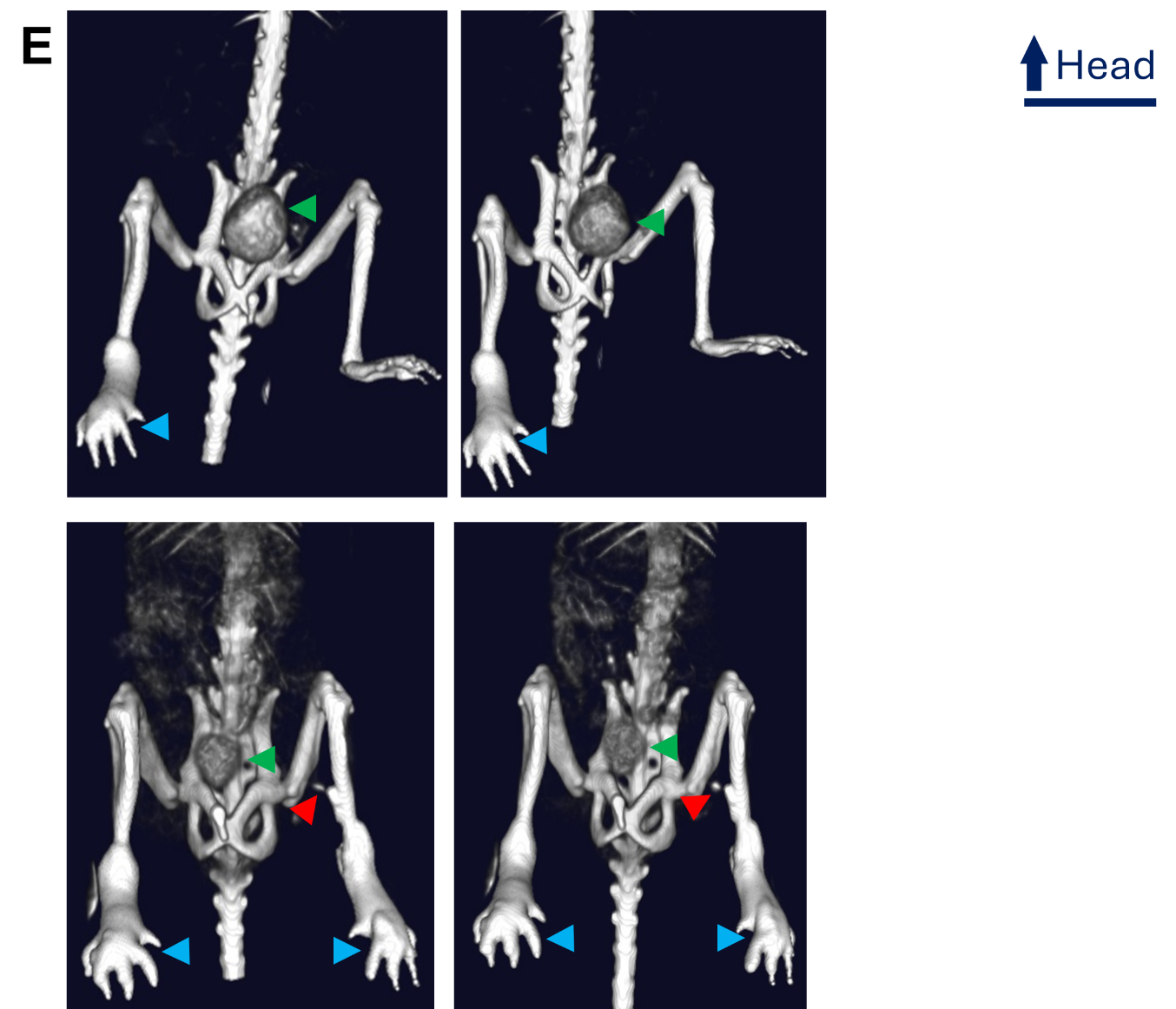
**

**Figure S8. Lymphatic tracing** **of lower limb region LNs (CT).** (A) Contrast agent was injected into the caudal mesenteric lymph node (LN). The flow of the contrast agent was recorded 5 min after injection at 5-min intervals. Blue arrowhead, injection site (caudal mesenteric LN); green arrowhead, right (R.) lateral iliac LN; red arrowhead, left (L.) lateral iliac LN. (B) Contrast agent injected into the left external iliac LN. The flow of the contrast agent was recorded 5 min after injection at 5-min intervals. Blue arrowhead, injection site (external iliac LN); red arrowhead, L. lumbar aortic LN; orange arrowhead, L. renal LN; green arrowhead, a trace of contrast agent. (C) Contrast agent injected into the right or left sciatic LN. The flow of the contrast agent was recorded 5 min after injection at 5-min intervals. Blue arrowhead, injection site (sciatic LN); green arrowhead, medial iliac LN; red arrowhead, lateral iliac LN; yellow arrowhead, renal LN. (D) Contrast agent injected into the right or left popliteal LN. The flow of contrast agent was recorded 5 min after injection at 5-min intervals. Blue arrowhead, injection site (popliteal LN); green arrowhead, sciatic LN; red arrowhead, lateral iliac LN; orange arrowhead, lumbar aortic LN; yellow arrowhead, renal LN; yellow arrow, efferent lymphatic vessels of popliteal LN. (E) Contrast agent was injected into the right or left hindfoot LN. The flow of contrast agent was recorded 5 min after injection at 5-min intervals. Blue arrowhead, injection site (hindfoot); green arrowhead, medial iliac LN; red arrowhead, popliteal LN.

**Table S1.** Body weight and organ weights of MXH10/Mo/lpr (*n* = 31) and MXH51/Mo/lpr (*n* = 9) mice.

| **Organs** | **MXH10/Mo/lpr** | | | **MXH51/Mo/lpr** | | | ***p* value** |
| --- | --- | --- | --- | --- | --- | --- | --- |
|  | **Mean** | **SEM** | ***n*** | **Mean** | **SEM** | ***n*** |  |
| Body weight (g) | 34.3 | 0.7 | 31 | 31.9 | 1.3 | 9 |  |
| R. SiLN (mg) | 227.0 | 21.5 | 31 | 242.1 | 48.1 | 9 |  |
| R. AALN (mg) | 186.9 | 19.5 | 31 | 281.2 | 30.8 | 9 |  |
| R. PALN (mg) | 194.8 | 17.9 | 31 | 270.4 | 33.9 | 9 |  |
| L. SiLN (mg) | 232.1 | 25.7 | 31 | 450.9 | 49.4 | 9 | 0.003 |
| L. AALN (mg) | 213.9 | 24.1 | 31 | 319.9 | 39.6 | 9 |  |
| L. PALN (mg) | 184.7 | 16.1 | 31 | 305.7 | 19.3 | 9 |  |
| Lung (mg) | 265.3 | 10.1 | 31 | 187.6 | 14.9 | 9 |  |
| Liver (mg) | 1675.6 | 52.4 | 31 | 1391.0 | 51.9 | 9 | <0.0001 |
| Heart (mg) | 137.1 | 3.9 | 31 | 108.6 | 6.0 | 9 |  |
| Kidney (mg) | 451.2 | 15.6 | 31 | 422.4 | 15.1 | 9 |  |
| Spleen (mg) | 657.1 | 72.9 | 31 | 248.8 | 22.2 | 9 | <0.0001 |
| Gl. submandibularis (mg) | 136.4 | 8.1 | 31 | 130.2 | 8.9 | 9 |  |
| Lymphoid organs (mg) | 254.2 | 17.3 | 31 | 281.2 | 18.6 | 9 |  |

Data are given as the mean ± SEM. **p* < 0.05, ***p* < 0.01, ****p* < 0.001, *****p* < 0.0001.

**Extended Video S1. Lymphatic flow imaging.** (A) *De novo* blood vessel observations from SiLN to PALN. (B) Lymph flow observations from the caudal mesenteric LN (provided as a separate MP4 file).
